# Genome-wide association study of extreme high bone mass: Contribution of common genetic variation to extreme BMD phenotypes and potential novel BMD-associated genes

**DOI:** 10.1016/j.bone.2018.06.001

**Published:** 2018-09

**Authors:** Celia L. Gregson, Felicity Newell, Paul J. Leo, Graeme R. Clark, Lavinia Paternoster, Mhairi Marshall, Vincenzo Forgetta, John A. Morris, Bing Ge, Xiao Bao, J.H. Duncan Bassett, Graham R. Williams, Scott E. Youlten, Peter I. Croucher, George Davey Smith, David M. Evans, John P. Kemp, Matthew A. Brown, Jon H. Tobias, Emma L. Duncan

**Affiliations:** a Musculoskeletal Research Unit, Translational Health Sciences, Bristol Medical School, University of Bristol, Bristol, UK; b Translational Genomics Group, Institute of Health and Biomedical Innovation, Queensland University of Technology at Translational Research Institute, 37 Kent Street, Woolloongabba 4102, QLD, Australia; c MRC Integrative Epidemiology Unit, University of Bristol, Bristol, UK; d Department of Human Genetics, McGill University and Genome Quebec Innovation Centre, Montreal, Quebec, Canada; e Lady Davis Institute, Jewish General Hospital, McGill University, Montreal, Quebec, Canada; f Molecular Endocrinology Laboratory, Department of Medicine, Imperial College London, Hammersmith Campus, London W12 0NN, UK; g The Garvan Institute of Medical Research, Sydney, New South Wales, Australia; h St Vincent's Clinical School, University of New South Wales Medicine, Sydney, New South Wales, Australia; i University of Queensland Diamantina Institute, Translational Research Institute, Brisbane, Queensland, Australia; j Royal Brisbane and Women's Hospital, Brisbane, Queensland, Australia

**Keywords:** Bone mineral density, *NPR3*, *SPON1*, Endochondral ossification, Wnt signalling

## Abstract

**Background:**

Generalised high bone mass (HBM), associated with features of a mild skeletal dysplasia, has a prevalence of 0.18% in a UK DXA-scanned adult population. We hypothesized that the genetic component of extreme HBM includes contributions from common variants of small effect and rarer variants of large effect, both enriched in an extreme phenotype cohort.

**Methods:**

We performed a genome-wide association study (GWAS) of adults with either extreme high or low BMD. Adults included individuals with unexplained extreme HBM (n = 240) from the UK with BMD Z-scores ≥+3.2, high BMD females from the Anglo-Australasian Osteoporosis Genetics Consortium (AOGC) (n = 1055) with Z-scores +1.5 to +4.0 and low BMD females also part of AOGC (n = 900), with Z-scores −1.5 to −4.0. Following imputation, we tested association between 6,379,332 SNPs and total hip and lumbar spine BMD Z-scores. For potential target genes, we assessed expression in human osteoblasts and murine osteocytes.

**Results:**

We observed significant enrichment for associations with established BMD-associated loci, particularly those known to regulate endochondral ossification and Wnt signalling, suggesting that part of the genetic contribution to unexplained HBM is polygenic. Further, we identified associations exceeding genome-wide significance between BMD and four loci: two established BMD-associated loci (5q14.3 containing *MEF2C* and 1p36.12 containing *WNT4*) and two novel loci: 5p13.3 containing *NPR3* (rs9292469; minor allele frequency [MAF] = 0.33%) associated with lumbar spine BMD and 11p15.2 containing *SPON1* (rs2697825; MAF = 0.17%) associated with total hip BMD. Mouse models with mutations in either *Npr3* or *Spon1* have been reported, both have altered skeletal phenotypes, providing in vivo validation that these genes are physiologically important in bone. *NRP3* regulates endochondral ossification and skeletal growth, whilst *SPON1* modulates TGF-β regulated BMP-driven osteoblast differentiation. Rs9292469 (downstream of *NPR3*) also showed some evidence for association with forearm BMD in the independent GEFOS sample (n = 32,965). We found *Spon1* was highly expressed in murine osteocytes from the tibiae, femora, humeri and calvaria, whereas *Npr3* expression was more variable.

**Conclusion:**

We report the most extreme-truncate GWAS of BMD performed to date. Our findings, suggest potentially new anabolic bone regulatory pathways that warrant further study.

## Highlights

• This GWAS found significant enrichment for associations with known BMD loci suggesting a polygenic contribution to high BMD
• We identified a novel locus at: 5p13.3 containing *NPR3* (lead SNP rs9292469) associated with lumbar spine BMD
• We identified a novel locus at: 11p15.2 containing *SPON1* (lead SNP rs2697825) associated with total hip BMD
• Mouse models with mutations in either *Npr3* or *Spon1* have been reported, both have altered skeletal phenotypes
• Our findings suggest potentially new anabolic bone regulatory pathways; however, further investigation is warranted

## 1 Introduction

Osteoporotic fractures are a major cause of morbidity and mortality, with associated healthcare costs exceeding $20 billion in the United States [1]. Understanding genetic regulation of bone signalling pathways, which underlie the pathogenesis of skeletal disease, aids development of novel therapeutics to increase bone mass [2]. Genome-wide association studies (GWAS) of bone density phenotypes, drawn mainly from general populations, have identified multiple BMD-associated loci, although together these only explain a relatively small proportion (5.8–11.8%) of variance in bone phenotypes [3, 4]. An alternative approach is to focus on rare individuals who represent extremes of a quantitative phenotype, i.e. BMD, to identify variants of relatively large effect. At the very extremes, high bone mass (HBM) and low bone mass (LBM) occur due to monogenic mutations (e.g. in *SOST*, *LRP5* or *LRP4* in HBM, and *COL1A1*, *COL1A2*, *LRP5* and others in LBM); however, such monogenic disorders fail to explain the vast majority of individuals with either HBM or LBM [5]. Conceivably, extreme HBM or LBM may both constitute polygenic conditions, either explained by variants in the same genes that determine BMD in the general population [3], or in novel extreme bone mass genes. In support of this, a previous GWAS of a moderate high and low BMD population replicated associations in 21 loci previously established to be BMD-associated from analyses of normal populations and identified six new genetic associations, highlighting the efficiency of extreme-truncated selection for quantitative trait GWAS design [6]. Such augmentation of statistical power through analysis of extreme phenotypes has been advantageous in a range of clinical phenotypes [[7], [8], [9], [10]] and is an established approach to investigate complex disease [11, 12].

Unexplained generalised HBM has a prevalence of 0.18% amongst a UK DXA-scanned adult population; affected individuals also have features suggestive of a mild skeletal dysplasia, such as mandibular enlargement and enthesophytes [13, 14]. We hypothesized that unexplained extreme HBM is genetically determined by variation in both established and novel BMD loci. In this GWAS of individuals with unexplained extreme HBM, we aimed to first determine whether variants in known loci account for BMD variation in this population. Secondly, we aimed to identify novel loci and validate the use of extreme BMD populations for genetic discovery. We augmented our unexplained extreme HBM population with a further moderate high BMD population and made comparison with an extreme-low BMD population to enhance statistical power. We investigated associated gene expression using human osteoblast expression quantitative trait loci (eQTL) and novel murine osteocyte expression data.

## 2 Methods

**Fig. 1:**
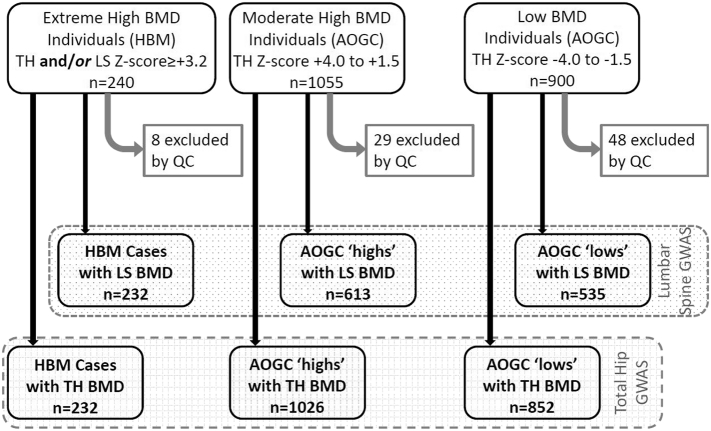
Flow diagram explaining recruitment of study populations with BMD data availability enabling lumbar spine (LS) and total hip (TH) GWAS (Stage 1). BMD; Bone Mineral Density. AOGC; Anglo-Australasian Osteoporosis Genetics Consortium. QC; Quality Control steps including exclusion of ethnic outliers.

We investigated three populations to identify genetic determinants of unexplained high BMD (Fig. 1, Supplementary Table 1). Individuals included: [1] UK-based unexplained extreme HBM index cases (n = 240) with total hip (TH) or first lumbar vertebra (L1) Z-score ≥+3.2; [2] more moderate high BMD females from the Anglo-Australasian Osteoporosis Genetics Consortium (AOGC) (n = 1055) with TH Z-scores between +1.5 and +4.0 [3] low BMD females also part of AOGC (n = 900), with TH Z-scores between −1.5 and −4.0, representing an extreme-low ‘super-control’ group, enhancing statistical power. Australian individuals who self-identify as Caucasian have been shown to be representative of UK populations regarding population stratification [6].

#### 2.1.1 High bone mass cases (Supplementary methods)

The HBM study is a UK based multi-centred observational study of adults with unexplained HBM, identified incidentally on routine clinical DXA scanning. Full details of DXA database screening and participant recruitment have previously been reported [13]. In brief, DXA databases containing 335,115 DXA scans were initially searched for a BMD T or Z-score ≥+4 at any site within the lumbar spine (LS) or hip, at UK 13 centres. All 1505 DXA images were visually inspected; 962 cases with established and/or artefactual causes of raised BMD were excluded, taking particular care to exclude osteoarthritic artefacts affecting the lumbar spine (see Supplementary Methods 1). A generalised HBM trait would be expected to affect both spine and hip BMD, though not necessarily to the same extent. Hence, we refined the definition of HBM index cases as a) L1 Z-score of ≥+3.2 plus TH Z-score of ≥+1.2 **and/or** b) TH Z-score ≥+3.2 plus L1 Z-score of ≥+1.2 (using age and gender-adjusted BMD Z-scores). A threshold of +3.2 was in keeping with the only published precedent for identifying HBM previously described using DXA [15], and these threshold combinations most appropriately differentiated generalised HBM from artefact. Z rather than T-score was used to limit age bias. Of 533 unexplained HBM index cases invited to participate, 248 (47%) were recruited between 2008 and 2010 [13], aged 18–90 years. After Sanger sequencing all HBM index cases, we excluded seven with *LRP5* mutations and one who carried a *SOST* mutation [5], leaving 240 unexplained HBM individuals for GWAS.

This study was approved by the Bath Multi-centre Research Ethics Committee (REC: 05/Q2001/78) and at each NHS Local REC.

#### 2.1.2 Anglo-Australasian Osteoporosis Genetics Consortium (AOGC) high BMD cases and low BMD controls (Supplementary methods)

The AOGC population included 1128 Australian, 74 New Zealand and 753 British women, aged between 55 and 85 years, five or more years postmenopausal, with either moderate high BMD (age and gender-adjusted BMD Z-scores of +1.5 to +4.0, n = 1055) or low BMD (age- and gender-adjusted BMD Z-scores of −4.0 to −1.5, n = 900) [6] (Fig. 1). Low BMD controls were excluded if they had secondary causes of osteoporosis (as previously described [6]).

The AOGC study was approved by the Queensland Office of Human Research Ethics Committee (Ref: 2008/018), the University of Queensland (Ref: 200800376) and/or relevant research ethics authorities at each participating centre. Some participants were recruited through genetic and/or clinical studies (all with appropriate ethical approval) but also provided written informed consent to contribute to collaborative genetic studies [[16], [17], [18]].

### 2.2 Genotyping and quality control

SNP genotyping was performed using Infinium OmniExpress-12v1.0 for the UK unexplained HBM cases (n = 240); and Illumina Infinium II HumHap300 (n = 140), 370CNVDuo (n = 4), 370CNVQuad (n = 1882) and 610Quad (n = 10) chips for the AOGC high and low BMD individuals (n = 2036), at the University of Queensland Diamantina Institute, Brisbane, Australia. For each study population genotype clustering was performed using Illumina's BeadStudio software; all SNPs with quality scores <0.15 and all individuals with <98% genotyping successes were excluded. Cluster plots from the 500 most strongly associated loci were manually inspected and poorly clustering SNPs excluded from analysis. Using PLINK, an IBS/IBD analysis was used to detect and exclude samples with cryptic relatedness [19]. SNPs with minor allele frequency (MAF) <1%, those with excess missingness (>95%) and those not in Hardy-Weinberg equilibrium (p < 1 × 10–6) were removed, leaving 181,323 SNPs in total shared across all chip types.

### 2.3 Population stratification

We used EIGENSTRAT software to detect population stratification, excluding 24 regions of long range linkage disequilibrium (LD) including the MHC, before running a principal component analysis using merged genotype data [20]. Eight unexplained HBM cases were removed as ethnic outliers (Fig. 1). Four eigenvectors, principal components of a genetic covariance matrix, were used as covariates to adjust for population stratification in all GWAS models.

### 2.4 Imputation

Imputation analyses were conducted for all datasets separately. Phasing was carried out using SHAPEIT [21] and imputation with IMPUTE2 [22], using a merged 1000G/UK10K reference dataset as the reference set of haplotypes, enabling imputation down to MAF 0.01. SNPs with high imputation quality (‘info score’ > 0.8) were included [23]; this stringent threshold was used as the two populations were imputed separately. All SNPs were filtered for missing data <0.05, HWE 1 × 10^−6^ and MAF > 0.01 (thus excluding rare HBM-causing monogenic disease). Additional concordance filtering was performed, excluding genotyped SNPs with r^2^ < 0.9 between original and masked IMPUTE2 imputed genotype, and those imputed SNPs in linkage disequilibrium (LD) (r^2^ > 0.2) with these excluded SNPs [24]. We further excluded known problematic UK10K imputed SNPs [25], and SNPs not present in both the 1000G and UK10K cohort imputation panels.

### 2.5 Stage 1: genome-wide association analysis

Association analyses for imputed genotypes were assessed with probabilistic genotypes under an additive (per allele) linear genetic model. Following imputation, 6,379,332 SNPs were tested for association in two quantitative trait analyses using the software SNPTEST. Quantitative analyses used genotyping data from individuals with UK unexplained HBM (n = 232), AOGC high BMD (n = 1026) and AOGC low BMD (n = 852) (Fig. 1), and tested association between SNPs and (i) TH-BMD Z-score (combined n = 2110) and (ii) LS-BMD Z-score (combined n = 1380). To explore whether genetic associations were specific to the very extremes of the BMD distribution, two additional quantitative analyses also tested these same associations restricted to the UK HBM cases (n = 232) and AOGC low BMD (n = 852) individuals again for (i) TH-BMD Z-score (n = 1084) and (ii) LS-BMD Z-score (n = 767). Quantitative models were adjusted for a priori covariates age, age^2^, study centre, and four eigenvectors. BMD Z-score was used as it standardises BMD by gender. Because gender was not evenly distributed within study populations (only 44 male individuals, all amongst unexplained HBM cases), inclusion as a covariate risks introducing sparse data bias [26], hence gender was not used as a covariate. The genomic inflation factors (λ) were 1.016 and 0.9973 for TH-BMD and LS-BMD respectively, and 1.037 and 1.016 in the restricted analyses (Supplementary Fig. 1). All LocusZoom association plots included both genotyped and imputed SNPs [27]. In pre-planned sensitivity analyses, all quantitative models were re-run with additional adjustment for height. Weight was not included as a covariate as increased android fat mass constitutes part of the unexplained HBM phenotype [28].

To establish whether BMD-increasing alleles were enriched in unexplained HBM, we performed a case-control GWAS of HBM cases versus unselected control individuals from the well described second Wellcome Trust Case Control Consortium (WTCCC2) (n = 5667) representing the general population, in whom BMD whilst unmeasured, is considered to be normal [29].

### 2.6 Stage 2: replication in GEFOS

We used publicly available data from the GEFOS 2015 meta-analysis (n = 32,965) of whole-genome sequencing, whole-exome sequencing, and deep imputation of genotype data (www.gefos.org/?q=content/data-release-2015) for SNPs associated with femoral neck (FN), LS and forearm BMD (chiefly distal radius measured), which had been adjusted for age, age^2^, gender and weight [30]. As the AOGC cohort had contributed to the GEFOS meta-data, the GEFOS 2015 meta-analysis was rerun excluding AOGC data (n = 30,970), with results used to assess replication of SNPs surpassing GWA significance (p < 5 × 10^−8^) in Stage 1.

### 2.7 Stage 3: gene expression

#### 2.7.1 Gene expression in primary human osteoblasts

To assess whether identified variants were involved in the regulation of messenger RNA levels via eQTLs, we performed cis-eQTL analyses of SNPs surpassing the GWA threshold (p < 5 × 10^−8^) in 95 primary human osteoblasts (as described previously [31]; GEO reference GSE15678), using genome-wide SNP data imputed to the combined UK10K and 1000G Phase 1 v3 reference panel [23]. Using α = 0.05 with Bonferroni correction, we aimed to identify gene targets for novel SNPs identified in Stage 1 (n = 4) and SNPs in LD (n = 31; r^2^ > 0.8), by examining gene expression profiles of all genes within 1 Mb of each lead SNP (included 24 gene probes).

#### 2.7.2 Gene expression in murine osteocytes

Osteocyte expression was determined through an analysis of whole transcriptome sequencing data from the primary osteocytes of four different bone types (tibia, femur, humerus and calvaria) from mice (marrow removed, 16 week old female mice, strain C57BL6/NTac, n = 8 per bone) [32]. RNA-sequencing reads were trimmed of low quality data using trimgalore [33], aligned to the GRCm38.p3 genome guided by the GENCODE M5 transcriptome annotation [34] using STAR [35] and expression quantified using RSEM [36]. A threshold of gene expression was determined based on the distribution of FPKM-normalised (Fragments Per Kilobase per Million mapped reads) gene expression for each sample [37]. “Expressed” genes were above this threshold for all 8 of 8 replicates in any bone type. Osteocyte enriched expression of these genes in the skeleton was determined by comparing transcriptome-sequencing data from bone-samples with osteocytes isolated versus those samples with marrow left intact (10-week old male mice, strain C57BL6/NTac, n = 5 per group) [32].

## 3 Results

### 3.1 Stage 1: analyses concerning established BMD-associated loci

**Fig. 2:**
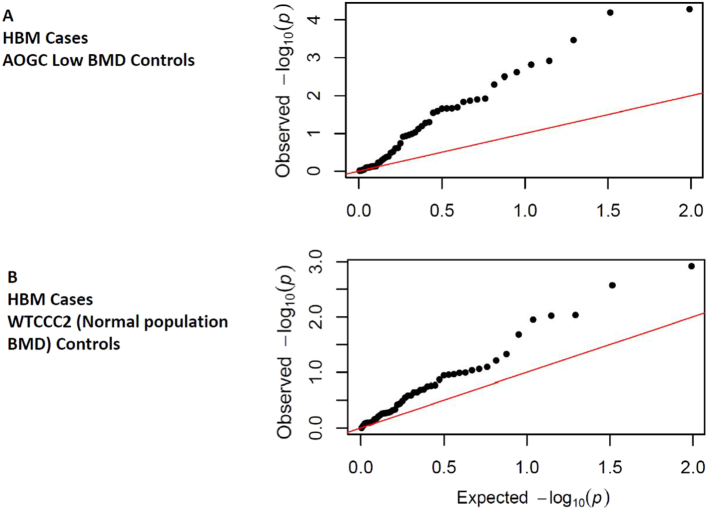
QQ plot for p values for 49 established BMD-associated loci in Case-Control GWAS of (A) unexplained HBM cases vs. AOGC low BMD controls, and (B) unexplained HBM cases vs. the second WTCCC2 (Wellcome Trust Case Control Consortium) controls. In both plots, the strength of observed associations for many SNPs far exceeded expected values.

In total, 49 of 64 SNPs previously associated at GWAS significance level with DXA BMD and/or fracture by Estrada et al. [3] were available in our imputed dataset (15 were not as they were either not imputed (n = 6), on chromosome X (n = 1), or were excluded by filtering for concordance (n = 7) or infoscore (n = 1)). The previously identified lead SNP at the *MEF2C* locus was associated with TH BMD at genome-wide significance (p < 5 × 10^−10^), and lead SNPs at *WNT4*/*ZBTB40*, *SOX6* and *CTNNB1* loci were strongly suggestive of association (p < 1 × 10^−6^). Overall, 29 of the available SNPs (59%) were associated with BMD at p < 0.05 (Supplementary Table 2). QQ plots of p values for the 49 tested SNPs assessing association at either TH or LS BMD (Supplementary Fig. 2) showed enrichment for known BMD-associated loci (i.e. observed p values were much smaller than those expected). However, to determine whether this enrichment is explained by excess variation in common BMD-increasing alleles and/or BMD-decreasing alleles, we ran two case-control GWAS' (Fig. 2). The first compared unexplained HBM cases against AOGC low BMD controls confirming the enrichment seen in quantitative GWAS. The second compared unexplained HBM cases against WTCCC2 controls considered to have normal BMD, confirming that unexplained HBM is polygenic reflecting enrichment at known common BMD-associated loci (Fig. 2).

As expected, βs for BMD measured at both the TH and LS were substantially larger in this HBM GWAS compared to the published general population GWAS from GEFOS [3]; all directions of effect were consistent (Supplementary Table 2), including when analysis was re-run including additional co-variates of gender and weight (Supplementary Table 3).

### 3.2 Stage 1: GWAS discovery findings

**Fig. 3:**
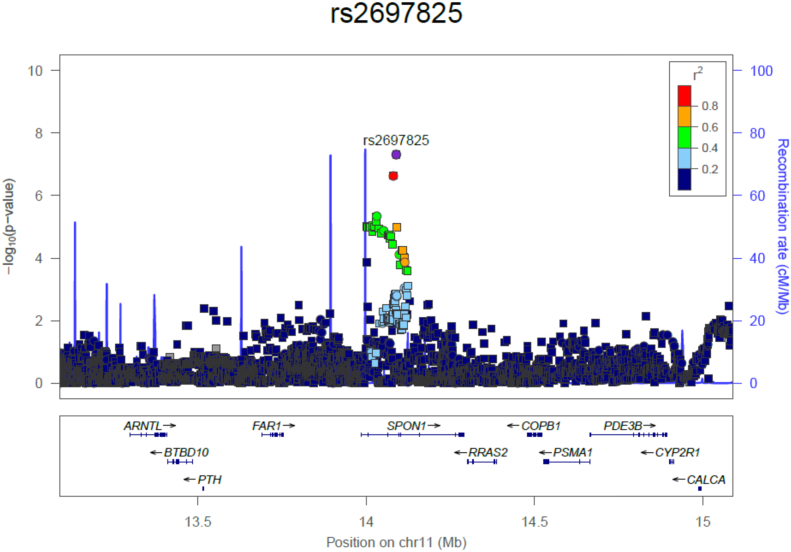
*SPON1* regional association plot of the unexplained HBM cases and AOGC Low BMD controls in a quantitative trait GWAS of total hip BMD Z-Score, adjusted for age, age^2^ and centre (1000 kb either side of rs2697825 shown). Square symbols indicate imputed SNPs; circles indicate those genotyped.

**Fig. 4:**
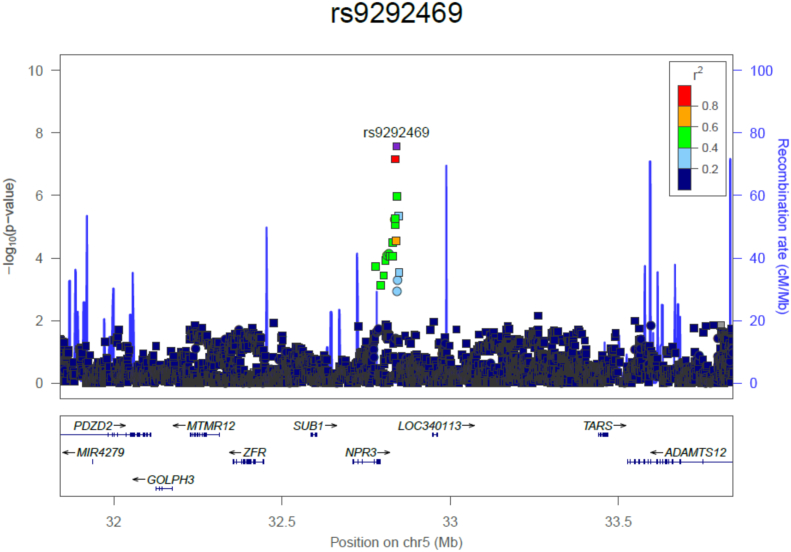
*NPR3* regional association plot of the unexplained HBM, AOGC High BMD cases and AOGC Low BMD controls in a quantitative trait GWAS of lumbar spine BMD Z-Score, adjusted for age, age^2^ and centre (1000 kb either side of rs9292469 shown). Square symbols indicate imputed SNPs; circles indicate those genotyped.

Our quantitative trait analyses identified SNPs at four loci that surpassed a genome-wide significant threshold (p < 5 × 10^−8^) (Table 1, Supplementary Fig. 3). Two loci, near *NPR3* and within *SPON1*, have not been implicated in GWAS of BMD previously, whereas *MEF2C* and *WNT4*/*ZBTB40* loci represent established BMD-associated regions [3] (Supplementary Fig. 4a & b). *MEF2C* and *WNT4*/*ZBTB40* loci were most strongly associated with TH-BMD Z-score, as was *SPON1* in analyses restricted to unexplained HBM and AOGC low-BMD individuals, suggesting this association may be specific to extreme BMD (rs2697825 lies within intron 3 of *SPON1*, Fig. 3). The *NPR3* locus was most strongly associated with LS-BMD Z-score (rs9292469 is 48.5 kb 3′ of *NPR3* with the LD block including part of this gene) (Fig. 4). As a SNP in the *NPR3* locus has previously been associated with adult height and truncal length [38], we re-ran our GWAS model with additional height adjustment; however, this did not attenuate our identified association (β 0.23, p = 2.25 × 10^−8^) (Supplementary Table 4, Supplementary Fig. 5). Height adjustment did however partially attenuate the association between rs2697825 (within the *SPON1* locus) and TH-BMD Z-score (Supplementary Table 4). We further identified 75 loci suggestive for association (p < 5 × 10^−5^) with TH-BMD and 71 with LS-BMD (Supplementary Tables 5 & 6). Results were unchanged in a sensitivity analysis excluding the 48 men (data not shown).

**Table 1:** Stage 1 - genome-wide significant SNPs with p < 5 × 10^−8^ associated with BMD Z-score measured at the total hip and lumbar spine.

rsID	Locus	Position	Closest gene/candidate	EA	EAF	Total hip BMD Z-score				Lumbar spine BMD Z-score			
						n	β	SE	p	n	β	SE	p
rs1366594	5q14.3	88376061	*MEF2C*	C	0.47	2110	−0.191	0.031	**4.83 × 10**^**−10**^	1380	−0.103	0.039	7.85 × 10^−3^
rs113784679	1p36.12	22648479	*WNT4*/*ZBTB40*	T	0.04	2110	0.511	0.088	**8.40 × 10**^**−9**^	1380	0.215	0.115	6.19 × 10^−2^
rs9292469	5p13.3	32840210	*NPR3*	T	0.33	2110	0.108	0.033	1.00 × 10^−3^	1380	0.232	0.041	**2.70 × 10**^**−8**^
rs2697825	11p15.2	14089431	*SPON1*	G	0.17	1084^a^	0.310	0.056	**4.93 × 10**^**−8**^	767^a^	0.188	0.065	3.92 × 10^−3^

Chromosome position using: GRCh37.p13.

Quantitative analysis results shown use data from HBM, AOGC high BMD and AOGC low BMD cohorts, except ^a^which reflects analyses restricted to HBM and AOGC low BMD cohorts. Model is adjusted for age, age^2^, centre and 4 PCs.

p < 5 × 10^−8^ appears in bold.

EA: Effect Allele (BMD increasing); EAF: Effect Allele Frequency. SE: Standard Error. β: effect estimate – represents change in BMD Z-score per copy of the SNP EA.

### 3.3 Stage 2: replication

The four SNPs identified in Stage 1 were assessed for replication using the GEFOS 2015 meta-analysis adjusted for age, age^2^, gender and weight, excluding AOGC data (Table 2). The *MEF2C* locus (rs1366594) replicated strongly in association with FN BMD, and to a lesser extent forearm BMD, but not with LS-BMD. The *WNT4*/*ZBTB40* locus (rs113784679) also replicated in association with FN BMD, and to a lesser extent LS-BMD. For both these loci the direction of association was concordant in the discovery and replication sets. The *NPR3* locus (rs9292469) showed no association with LS-BMD, but, although not withstanding correction for testing 4 SNPs, was weakly associated with BMD measured at the forearm (p = 0.0198) and FN (p = 0.06), the direction of association being concordant with the discovery set. The *SPON1* locus (rs2697825) was associated weakly (not withstanding multiple testing correction) with BMD measured at the forearm (p = 0.029); however, the direction of effect was discordant, and no association was seen at the LS or FN. Unfortunately, non-weight adjusted GEFOS GWAS meta-data were not available for replication.

**Table 2:** Stage 2 - genome-wide significant SNPs with p < 5 × 10^−8^ associated with BMD Z-score measured at the total hip and lumbar spine and replication in GEFOS.

rsID	Closest gene/candidate	EA	EAF	Stage 1: total hip BMD Z-score		Stage 1: lumbar spine BMD Z-score		GEFOS EAF	Stage 2: GEFOS excluding AOGC FN BMD		Stage 2: GEFOS excluding AOGC LS BMD		Stage 2: GEFOS excluding AOGC Forearm BMD	
				Β	p	β	p		β	p	β	p	β	p
rs1366594	*MEF2C*	C	0.47	−0.191	**4.83 × 10**^**−10**^	−0.103	7.85 × 10^−3^	0.47	−0.073	**2.93 × 10**^**−20**^	−0.003	0.77	−0.077	**3.40 × 10**^**−6**^
rs113784679	*WNT4*/*ZBTB40*	T	0.04	0.511	**8.40 × 10**^**−9**^	0.215	6.19 × 10^−2^	0.04	0.080	**1.89 × 10**^**−4**^	0.058	**0.022**	−0.002	0.97
rs9292469	*NPR3*	T	0.33	0.108	1.00 × 10^−3^	0.232	**2.70 × 10**^**−8**^	0.35	0.015	0.060	0.002	0.84	0.040	**0.0198**
rs2697825	*SPON1*	G	0.17	0.310 ^a^	**4.93 × 10**^**−8**^	0.188 ^a^	3.92 × 10^−3^	0.18	0.006	0.55	0.011	0.34	−0.046	**0.0293**

Chromosome position using: GRCh37.p13.

Quantitative analysis results shown use data from HBM, AOGC high BMD and AOGC low BMD cohorts, except ^a^which reflects analyses restricted to HBM and AOGC low BMD cohorts. Stage 1 model adjusted for age, age^2^, centre and 4 PCs. Stage 2 model GEFOS adjusted for age, age^2^, gender and weight.

Stage 1 p < 5 × 10^−8^ appears in bold. Stage 2 p < 0.05 appears in bold.

EA: Effect Allele (BMD increasing); EAF: Effect Allele Frequency. BMD: Bone Mineral Density. FN: Femoral Neck. LS: Lumbar Spine. SE: Standard Error. β: effect estimate – represents change in BMD Z-score per copy of the SNP EA.

### 3.4 Stage 3: gene expression

#### 3.4.1 Gene expression in primary human osteoblasts

We tested the association of potential functional SNPs with cis-eQTL expression of genes in human osteoblasts from 95 donors [31] in whom genotype data were available and imputed to a combined UK10K/1000G reference panel [23]. We identified potential target genes based on cis-eQTL evidence in human osteoblasts for two of four of our lead SNPs (Supplementary Table 7). Whilst rs113784679 was identified as having a potential target gene of *WNT4* (p = 0.002), rs1366594 (*MEF2C* nearest gene) had no evidence of a target gene in osteoblasts. eQTL data did not support a specific gene association for rs9292469 (*NPR3* nearest gene) (osteoclast and chondrocyte expression data were not available). SNPs in LD with rs2697825, which lies within an intron (3/16) of *SPON1*, were associated with *BTBD10* expression after Bonferroni correction (Supplementary Tables 8 & 9). *BTBD10* has not previously been associated with bone regulation. We assessed all SNPs lying 500 kb either side of *BTBD10* in GEFOS 2015 meta-analysis summary data; using ANNOVAR for SNP annotation 66,654 SNPs were assessed for association with BMD (at FN, LS and forearm) [39], but no evidence of association was detected (all p > 5 × 10^−4^).

#### 3.4.2 Expression of Spon1 and Npr3 in murine osteocytes

*Spon1* and *Npr3* were investigated as the two genes lying closest to SNPs identified in Stage 1. *Spon1* was highly expressed in osteocytes in all four bone types forming part of a characteristic “osteocyte signature”, which is a list of genes significantly enriched in osteocytes relative to other cells in the marrow space and actively expressed in every replicate (Table 3). *Npr3* was expressed in osteocytes although at much lower levels than *Spon1* and despite some variability it was actively expressed in all tibia and 7 of 8 humeri samples. Considering the osteoblast eQTL results, we also examined *Btbd10* expression. *Btbd10* was expressed in whole femur, tibia, humerus and skull but its expression was not enriched in osteocytes.

**Table 3:** Stage 3 - osteocyte expression by whole transcriptome sequencing of Npr3 and Spon1 in four bone types (tibia, femur, humerus and calvaria) from mice.

MGI gene symbol	Chrom	Skeletal GO	Tibia		Femur		Humerus		Calvaria		Clean Bone		Bone And Marrow		Expressed in all bone types	Enriched in osteocyte samples	Osteocyte signature
			Activity	Mean FPKM	Activity	Mean FPKM	Activity	Mean FPKM	Activity	Mean FPKM	Activity	Mean FPKM	Activity	Mean FPKM			
Npr3	15	GO:0001501 GO:0002158 GO:0033688	Active 8/8	1.14	Active 2/8	0.52	Active 7/8	1.57	Inactive	0.24	Active 5/5	1.59	Inactive	0.21	No	Yes	No
Spon1	7	na	Active 8/8	12.0	Active 8/8	7.17	Active 8/8	11.0	Active 8/8	3.74	Active 5/5	7.57	Active 5/5	1.46	Yes	Yes	Yes

MGI: Mouse Genome Informatics.

Mouse Ensembl Ids: *Npr3* - ENSMUSG00000022206; *Spon 1* - ENSMUSG00000038156.

Skeletal GO: Gene Ontology (http://www.ebi.ac.uk).

Activity: Number of replicates of that bone type with FPKM expression values above active gene threshold.

Clean bone: bone with marrow removed to isolate osteocytes.

Bone and marrow: bone cleaned of connective tissue and growth plates with the marrow left intact.

Mean FPKM: Mean gene expression in for gene across samples of bone type normalised for gene length and library size (Fragments Per Kilobase per Million mapped reads).

## 4 Discussion

Firstly, we have identified over-representation of signal from known BMD loci, implying that unexplained HBM is, at least in part, polygenic in origin, influenced by common genetic variation. Secondly, our GWAS has identified two genome-wide significant SNPs, rs9292469 (3′ of *NPR3*) and rs2697825 (within an intron of *SPON1*), and confirmed two established BMD-associated loci, *MEF2C* and *WNT4*/*ZBTB40*, despite our limited sample size. Replication of rs9292469 was only weak (concordant at distal forearm, rather than LS) and for rs2697825 was discordant for both site (distal forearm, rather than hip) and direction. However, two genes at these loci are expressed in bone, and mutations of these genes in mice have been shown to influence bone mineral content (BMC) (see below), giving support to these as novel HBM-associated genes, although further investigation is warranted.

### 4.1 Established BMD-associated loci

Of the four BMD-associated loci published by Estrada et al. and identified in our analysis with p < 5 × 10^−5^, both *MEF2C* (for which we found the strongest evidence of association) and *SOX6* regulate endochondral ossification, linking this pathway to high BMD. *MEF2C* controls chondrocyte hypertrophy, cartilage ossification, and longitudinal bone growth in mice [40]. In addition to its role in bone mass accrual during skeletal growth, endochondral ossification is involved in the formation of osteophytes and enthesophytes [[41], [42], [43]], both prevalent in unexplained HBM [14]. This raises the possibility that variation in endochondral ossification pathway genes may be particularly relevant in HBM, contributing pleiotropically to both higher BMD and the wider associated skeletal phenotype. Besides *WNT4*, several SNPs annotated to Wnt/β-catenin signalling pathway genes (*MEF2C*, *CTNNB1*, *RSPO3*, and *WLS*), suggesting enhanced osteoblast activity may also contribute to the HBM phenotype.

### 4.2 NPR3 (natriuretic peptide receptor 3)

The transmembrane protein NPR3 acts as a clearance receptor modulating C-type natriuretic peptide (CNP) activity [37, 38]. The importance of natriuretic peptide (NP) signalling in bone is increasingly recognised. Natriuretic peptides ANP, BNP and CNP bind to three NP receptors (NPRs): ANP and BNP preferentially bind to NPR1; NPR2 to CNP; and NPR3 to all three NPs with similar affinity. Additionally, osteocrin, a protein secreted by osteoblast-lineage cells with homology to NPs, also binds to NPR3 and can displace CNP, with subsequent enhanced signalling through NPR2 [44]. NPR2 and NPR3 are both expressed in chondrocytes and osteoblasts [28, 37]. Disrupted NP signalling results in marked skeletal phenotypes, with evidence from both mouse and human data.

In humans, enhanced signalling results from autosomal dominant activating *NPR2* mutations causing bony overgrowth and tall stature [41] (MIM# 615923). CNP overproduction causes bony overgrowth and childhood skeletal abnormalities, particularly affecting stature, vertebrae and digital length [[33], [34], [35], [36]] (MIM# 600296). Mice overexpressing BNP or CNP have elongated bones [45, 46], as do those overexpressing osteocrin, where NPR3 clearance of CNP is reduced, resulting in increased NPR2 signalling [44]. Multiple spontaneous and ENU (*N*-ethyl-*N*-nitroso urea) mouse strains with *Npr3* mutations have skeletal phenotypes with increased linear growth, bone area and kyphosis, as endochondral ossification is delayed expanding the growth plate [[47], [48], [49], [50]]. BMC is increased in female, but not male homozygous ENU mice, but as bone size is also increased, BMD is normal [48]. A single histological section, which requires further validation, has suggested trabeculae may be thicker and longer [49]. This skeletal overgrowth due to impaired endochondral ossification is thought to result from increased activation of p38 MAPK signalling [[47], [48], [49]], which presumably delays growth plate quiescence.

In contrast, in humans attenuated NP signalling from loss-of-function *NRP2* mutations cause autosomal recessive acromesomelic dysplasia with extreme short stature [39] (MIM# 602875); heterozygous carriers display reduced height [40] (MIM# 616255). Whilst in mice lacking CNP or with loss-of-function *Npr2* mutations exhibit impaired longitudinal bone growth [51, 52].

In healthy adolescent humans, cross-sectional studies associate CNP synthesis with pubertal linear growth in, supporting its role in endochondral ossification [53]. CNP is a weak natriuretic [54]; the role of CNP/NRP3 regulation in explaining the hyponatraemia-low bone mass association [55] is unknown. A GWAS identified association between an *NPR3* SNP (rs10472828) and adult height and truncal length [38]; importantly additional height-adjusted GWAS did not alter our findings. Of note, rs9292469 was associated with heel Broadband Ultrasound Attenuation (BUA) in the discovery phase of a GWAS of 14,258 participants (p = 3.1 × 10^−6^); this finding was not replicated by meta-analysis [56].

*NPR3* may be the target gene regulated by rs9292469, as our human osteoblast eQTL data do not support an alternative target, and we and others have shown *Npr3* expression in the mouse skeleton [48]. Previous findings in our unexplained HBM population have shown that although adult height is no different from controls, trabeculae are thicker increasing trabecular density [57, 58], possibly recapitulating observations in *Npr3*-mutant mice [47, 49]. Our previous analyses suggested unexplained HBM protects against age-associated declines in trabecular BMD [57]. It is interesting that association was seen at the lumbar spine, and weakly replicated at the distal forearm (although not the LS); both are ‘trabecular-rich’ sites. Taken together, our findings are consistent with previous observations of the role of NPR3 in regulating skeletal growth.

### 4.3 SPON1 (Spondin 1/F-spondin)

rs2697825 is an intronic variant in *SPON1*. *SPON1*, coding for an extracellular matrix glycoprotein, has not previously been associated with a bone phenotype in humans. *Spon1* knockout mice have a skeletal phenotype consistent with HBM, with *Spon1*^*−/−*^ mice at 6 months age having 60% higher bone volume, bone volume/total volume, cortical area and trabecular number with reduced trabecular spacing [59]. Increased trabecular density and reduced endosteal expansion is seen in human HBM [57, 58]. Serum CTX-1 and TRAP levels are normal in *Spon1*^*−/−*^ mice; however, TGF-β1 levels are reduced compared with WT mice, whilst SMAD 1/5 activation is enhanced in both osteoblasts and chondrocytes [59]. Although vertebrae and intramembranous bones have not been characterized in *Spon1*^*−/−*^ mice, overall findings suggest reduced F-spondin activity decreases TGF-β1 levels, permitting activation of SMAD family transcription factors which, in conjunction with RUNX-2, promote BMP-driven osteoblast differentiation and hence bone formation [60]. In addition to our observed osteocyte expression, *SPON1* expression is evident in several musculoskeletal tissues: the embryonic growth plate cartilage, periodontal tissue and human and rodent osteoarthritic cartilage where *SPON1* expression appears to activate TGF-β, inducing cartilage degradation [[61], [62], [63]]. Surprisingly, our eQTL results suggested *BTBD10* rather than *SPON1* may be the target gene for rs2697825. *BTBD10* (Broad-Complex Tramtrack Domain Containing 10) activates AKT by phosphorylation in neuronal and pancreatic beta cells. *BTBD10* overexpression accelerates growth of pancreatic beta cells [64]. Despite expression in bone, *BTBD10* has no known role in bone regulation.

The relatively high frequency of the four identified SNPs (MAFs 0.04–0.47), compared to the rarity of the HBM phenotype, raises the possibility that these common variants are in LD with rare high-effect variants. Some candidate studies in type 1 diabetes and Alzheimer's disease identified stronger effects from rare variants than the common variants responsible for the initial identification of the associated gene(s) [65, 66]. However, this is not a universal finding; we note the relative paucity of low frequency high-effect variants in a recent fractures analysis in 508,253 individuals [30]. No associations of rare variants in *NPR3* and *SPON1* with BMD have been reported to date. Segregation studies show the majority of BMD heritability is polygenic [[67], [68], [69], [70], [71]]. In specific populations, monogenic effects may be observed, but always on a polygenic background [68, [71], [72], [73]]. The extent to which variation associated with *NPR3* and *SPON1* interacts with the enriched background polygenic architecture in HBM is unclear; we lacked power to assess gene-gene interactions. However, our ability to detect association with *NPR3* and *SPON1* loci, despite our relatively small sample size, is testament to their likely effect sizes in this unusual population.

GWAS of the AOGC cohort alone has been published previously [6]; however, associations with *NPR3* and *SPON1* loci were not identified. Only 59% of the AOGC have LS BMD available, so adding 232 more extreme HBM cases, in whom artefactual elevations in BMD were excluded, substantially increased statistical power. Moreover, the *SPON1* locus was identified in analyses restricted to 232 unexplained HBM (+AOGC low-BMD) individuals, who arguably represent a more precisely defined and extreme population.

### 4.4 Limitations

The failure to replicate the associations of these genes at either LS or FN in the GEFOS (minus AOGC) cohort suggests that either there are differences in the genetic structure determining BMD in the extreme discovery cohort compared with the general population; alternately, these findings are false positives or the failure to replicate is a false negative, due to statistical power issues or differences in covariate handling. That the *SPON1* locus was identified in analyses restricted to a more precisely defined and extreme population supports variation at this locus being more specific to HBM individuals; however, this was not the case for the *NPR3* locus. Given the large size of the GEFOS cohort, failure to replicate even at nominal levels of significance, indicates that at the very least these variants do not have the same effect size in the general population as observed here. Distinguishing between these explanations requires further studies in extreme bone mass cohorts, in much larger BMD association databases, or in genetically modified animals. Unfortunately, no second extreme BMD population currently exists, hence our use of a large general population dataset of DXA-measured BMD. It was not possible to re-run a non-weight adjusted GWAS for BMD across all GEFOS cohorts for meta-analysis. Comparisons between our study and those reported in the general population are hindered by these differences; we were reluctant to adjust for weight as this a known feature of unexplained HBM [28]. Generalisability of our findings is limited to Caucasian females, given the small number of men in this study. Genotype data came from a range of platforms which introduces heterogeneity and thus could have biased the discovery results. Our use of stringent ‘information score’ thresholds and concordance filters may also have missed true associations. Lastly, whilst we included both osteocyte and osteoblast expression data, osteoclast and chondrocyte expression data were lacking.

## 5 Conclusions

Common variation in established BMD genes is over-represented in unexplained HBM, suggesting HBM is, at least in part, polygenic in origin with contribution from the same genes that determine BMD in the general population. Functional annotation suggested that genes particularly contributing to the HBM phenotype are involved in endochondral ossification and osteoblast differentiation and activity. Two novel BMD-associated loci have been identified with candidate genes *NRP3* and *SPON1*, associated with lumbar spine and hip BMD respectively. These findings are supported by *NRP3* and *SPON1* bone expression data; further, both *Npr3* and *Spon1* have reported mouse models with altered skeletal phenotypes providing biological validation that these genes play a functional role in bone. Whilst small, our GWAS results are certainly hypothesis-generating and highlight potentially new anabolic bone regulatory pathways which warrant further study.

## Acknowledgments (see Supplementary data)

We thank GEFOS and the Gene Expression Omnibus (GSE54461) for their publicly available data.

## Funding

CLG was funded by the Wellcome Trust (080280/Z/06/Z), the EU 7th Framework Programme under grant agreement number 247642 (GEoCoDE), a British Geriatric Society travel grant, and Arthritis Research UK (grant ref. 20000). This study was supported by the NIHR CRN (portfolio number 5163). The AOGC was funded by the National Health and Medical Research Council (Australia) (grant ref. 511132). GRW, JHDB and PIC are funded by a Wellcome Trust Strategic Award (grant ref. 101123). LP and DE work in a unit which receives UK Medical Research Council funding (MC_UU_12013/4).

## Author contributions

Study design CG, PL, DB, GW, SY, PC, GDS, MB, JH, ED; Study conduct CG, MA, JT, ED; Data collection CG; Data analysis CG, FN, PL. LP, MM, GC, JM, XB, SY, PC; Data interpretation CG, FN, LP, JM, SY, PC, DE, JK, MB, JT, ED; Drafting manuscript CG, JT, ED; Revising manuscript CG, LP, JM, DB, GW, SY, PC, DE, JK, MB, ED. Approving final version CG, LP, JK, JT, ED. CG, FN, PL, and ED take responsibility for the integrity of the data analysis.

## Appendix A Supplementary data

Supplementary material (supplementary methods, supplementary tables 1 to 9, supplementary acknowledgements) Image 1

Supplementary figures 1 to 5 Image 2
