# Supplementary material for: Genome-wide association study of extreme high bone mass: Contribution of common genetic variation to extreme BMD phenotypes and potential novel BMD-associated genes
Source: Bone. 2018 Sep;114:62–71. doi: 10.1016/j.bone.2018.06.001 (PMC6086337; doi:10.1016/j.bone.2018.06.001)
Supplement: Supplementary file 1 — Supplementary material (supplementary methods, supplementary tables 1 to 9, supplementary acknowledgements) [file mmc1.docx]

**Supplementary Materials for ‘****Genome-wide association study of extreme high bone mass: contribution of common genetic variation to extreme BMD phenotypes and potential novel BMD-associated genes’**

**Gregson et al**

**Contents:**

Supplementary Methods: High Bone Mass Cases and Anglo-Australasian Osteoporosis Genetics Consortium (AOGC)

Supplementary Table 1: Study descriptives for the populations contributing to quantitative trait GWAS

Supplementary Table 2: (Stage 1: Analyses concerning established BMD-associated loci) Results for 64 established BMD-associated loci published by Estrada et al, showing associations for HBM GWAS of total hip and lumbar spine, adjusting for age, age^2^, center and population stratification

Supplementary Table 3: (Stage 1: Analyses concerning established BMD-associated loci) GWAS results for 64 established BMD-associated loci published by Estrada et al, showing associations for HBM GWAS of total hip and lumbar spine, adjusting for age, age^2^, center, gender, weight and population stratification

Supplementary Table 4: (Stage 1: GWAS discovery) Sensitivity Analysis: Genome-wide significant SNPs with p<5x10^-8^ associated with BMD Z-score measured at the total hip and lumbar spine after additional height adjustment

Supplementary Table 5: (Stage 1: GWAS discovery) 75 SNPs with p<5x10-5 associated with BMD Z-score measured at the total hip in Stage 1 GWAS

Supplementary Table 6: (Stage 1: GWAS discovery) 71 SNPs with p<5x10-5 associated with BMD Z-score measured at the lumbar spine in Stage 1 GWAS

Supplementary Table 7: (Stage 3: Gene expression) Results from *cis*-eQTL analyses in primary osteoblast samples taken from 95 human donors for four lead SNPs which reached GWAS significance

Supplementary Table 8: (Stage 3: Gene expression) Results from *cis*-eQTL analyses in primary osteoblasts for rs9292469 and SNPs in Linkage Disequilibrium

Supplementary Table 9: (Stage 3: Gene expression) Results from *cis*-eQTL analyses in primary osteoblasts for rs2697825 and SNPs in Linkage Disequilibrium

Acknowledgements

References

**Supplementary Methods 1:**

**High Bone Mass cases**

DXA databases containing 335,115 DXA scans were initially searched for individuals with a BMD T or Z-score ≥+4 at any site within the lumbar spine (LS) or hip, at 13 centers in England and Wales (9 Hologic, 4 Lunar). A further two centers with Hologic scanners contributed 23 similar individuals identified prospectively. All 1505 DXA images were visually inspected; 962 cases with significant osteoarthritis and/or other causes of raised BMD were excluded (*e.g*. surgical metalwork, Paget’s disease, metastases (1)). Evidence of significant osteoarthritis (OA) on lumbar DXA scans is common. To reduce contamination of remaining DXA scans by more moderate OA, we refined case definition by restricting analyses to specific lumbar vertebra(e). At the largest center, 562 scans with T/Z-score ≥+4 were graded for OA severity and examined in relation to BMD at lumbar vertebral levels (2, 3). In contrast to other lumbar vertebrae, L1 Z-score was not associated with OA, reflecting the recognized pattern of progressive OA changes seen in descending sequential lumbar vertebrae (4). Further, presence of high TH BMD did not correlate with co-existent LS OA.

A standard deviation of +3.2 would be expected to identify a tail of 0.069% of a normal distribution (5).

Of 533 unexplained HBM index cases invited to participate, 240 (45%) were recruited between September 2008 and April 2010 (6). Written informed consent was collected for all in line with the Declaration of Helsinki (7). Participants were excluded if under 18 years of age, pregnant or unable to provide written informed consent. All participants were assessed using a standardised structured history and examination questionnaire. DNA was extracted from peripheral venous blood using standard phenol/chloroform extraction techniques.

**Anglo-Australasian Osteoporosis Genetics Consortium (AOGC)**

BMD Z-scores were determined according to the Geelong Osteoporosis Study normative range (8).

Low BMD controls were excluded if they had secondary causes of osteoporosis, including corticosteroid usage at doses equivalent to prednisolone ≥7.5 mg/day for ≥6 months, past or current anticonvulsant usage, previous strontium usage, premature menopause (<45 years), alcohol excess (>28 units/week), chronic renal or liver disease, Cushing’s syndrome, hyperparathyroidism, thyrotoxicosis, anorexia nervosa, malabsorption, coeliac disease, rheumatoid arthritis, ankylosing spondylitis, inflammatory bowel disease, osteomalacia, and neoplasia (cancer, other than skin cancer). Screening blood tests (including creatinine (adjusted for weight), alkaline phosphatase, gamma glutamyltransferase, 25-hydroxyvitamin D and PTH) were checked in 776 cases, and no differences were found between the high and low BMD groups. Therefore, no further screening tests were done for the remaining cases.

DNA was obtained from peripheral venous blood from all cases except those recruited from New Zealand, for whom DNA was obtained from salivary samples using Oragene kits (DNA Genotek, Ontario, Canada). We previously demonstrated that DNA from these two sources has equivalent genotyping characteristics (9). Recruited participants gave written, informed consent.

**Supplementary Table 1: Study descriptives for the populations contributing to quantitative trait GWAS**

|  | **HBM cases** | **AOGC**  **high BMD** | **AOGC**  **low BMD** |
| --- | --- | --- | --- |
| Total n | 240 | 1055 | 900 |
| Female, n (%) | 192 (80%) | (100%) | (100%) |
| Age (years) | 60.2 (11.1) | 70.4 (8.3) | 68.7 (8.8) |
| Height (cm) | 165.8 (8.1) | 160.5 (6.2) | 158.8 (7.5) |
| Weight (kg) | 85.8 (17.6) | 77.5 (14.7) | 61.5 (13.7) |
| Total hip Z-score^a^ | +3.27 (1.05) | +2.04 (0.50) | -1.92 (0.40) |
| Lumbar spine Z-score^b^ | +4.91 (1.33) | +2.20 (1.62) | -1.05 (1.08) |

HBM: High Bone Mass. AOGC: Anglo-Australasian Osteoporosis Genetics Consortium

Mean (SD) shown for continuous variables which were normally distributed.

^a^ Maximal total hip of right/left

^b^ Either L1-4 or L2-4 used depending upon center availability

**Supplementary Table 2: (Stage 1: Analyses concerning established BMD-associated loci) Results for 64 established BMD-associated loci published by Estrada et al, showing associations for HBM GWAS of total hip and lumbar spine, adjusting for age, age^2^, center and population stratification**

|  |  |  |  |  |  | **Estrada et al**  **FN** | | **HBM_AOGC**  **Total Hip** | | **Estrada et al**  **LS** | | **HBM_AOGC**  **LS** | | |
| --- | --- | --- | --- | --- | --- | --- | --- | --- | --- | --- | --- | --- | --- | --- |
| **SNPID** | **LOCUS** | **POSITION** | **GENE** | **EA** | **EAF** | **FN-B** | **FN-P** | **HIP-B** | **HIP-P** | **LS-B** | **LS-P** | **LS-B** | **LS-P** | |
| **rs1366594** | **5q14.3** | **88411817** | ***MEF2C*** | **A** | **0.54** | **0.08** | **4.00E-61** | **0.19** | **4.83E-10** | **0.01** | **1.00E-02** | **0.10** | **7.85E-03** | |
| **rs6426749** | **1p36.12** | **22584060** | ***ZBTB40/WNT4*** | **C** | **0.17** | **0.11** | **7.00E-57** | **0.21** | **2.36E-07** | **0.1** | **1.80E-44** | **0.14** | **4.43E-03** | |
| **rs7108738** | **11p15.2** | **15666660** | ***SOX6*** | **T** | **0.83** | **-0.08** | **1.00E-32** | **-0.20** | **7.78E-07** | **-0.03** | **2.14E-06** | **-0.19** | **6.68E-05** | |
| **rs430727** | **3p22.1** | **41103568** | ***CTNNB1*** | **T** | **0.48** | **-0.06** | **4.00E-25** | **-0.15** | **9.10E-07** | **-0.05** | **1.50E-18** | **-0.12** | **1.24E-03** | |
| rs13204965 | 6q22.33 | 127208765 | *RSPO3* | A | 0.76 | 0.04 | 8.00E-12 | 0.14 | 1.14E-04 | 0.04 | 3.60E-10 | 0.10 | 2.83E-02 | |
| rs1346004 | 2q24.3 | 166309292 | *GALNT3* | A | 0.50 | -0.05 | 1.00E-25 | -0.12 | 1.74E-04 | -0.06 | 4.00E-30 | -0.05 | 1.77E-01 | |
| rs12407028 | 1p31.3 | 68420304 | *WLS* | T | 0.60 | 0.06 | 3.40E-23 | 0.12 | 1.91E-04 | 0.08 | 3.00E-45 | 0.09 | 2.15E-02 | |
| rs7851693 | 9q34.11 | 132468648 | *FUBP3* | C | 0.64 | 0.05 | 3.00E-22 | 0.11 | 4.62E-04 | 0.03 | 6.00E-08 | 0.11 | 5.00E-03 | |
| rs4796995 | 18p11.21 | 13698574 | *FAM210A* | A | 0.63 | 0.03 | 5.00E-08 | 0.11 | 5.43E-04 | 0.02 | 6.65E-04 | 0.05 | 1.90E-01 | |
| rs7584262 | 2p21 | 42104053 | *PKDCC* | T | 0.23 | 0.04 | 1.00E-09 | 0.13 | 6.23E-04 | 0.01 | 7.00E-02 | 0.05 | 3.14E-01 | |
| rs4727338 | 7q21.3 | 95958611 | *SHFM1* | C | 0.67 | 0.08 | 8.00E-48 | 0.11 | 9.05E-04 | 0.07 | 2.00E-35 | 0.11 | 8.03E-03 | |
| rs3801387 | 7q31.31 | 120762001 | *WNT16* | A | 0.74 | -0.08 | 5.00E-40 | -0.11 | 1.12E-03 | -0.09 | 3.00E-51 | -0.08 | 6.04E-02 | |
| rs10416218 | 19q13.11 | 38290967 | *GPATCH1* | T | 0.73 | -0.03 | 5.50E-08 | -0.11 | 1.24E-03 | -0.04 | 7.00E-11 | -0.07 | 1.23E-01 | |
| rs9533090 | 13q14.11 | 41849449 | *AKAP11* | T | 0.49 | -0.05 | 5.00E-23 | -0.10 | 1.30E-03 | -0.10 | 5.00E-68 | -0.14 | 2.20E-04 | |
| rs9466056 | 6p22.3 | 21492592 | *CDKAL1* | A | 0.38 | -0.03 | 3.00E-08 | -0.10 | 1.47E-03 | -0.04 | 3.00E-13 | -0.09 | 2.39E-02 | |
| rs2062377 | 8q24.12 | 120076601 | *TNFRSF11B* | A | 0.57 | -0.06 | 9.00E-25 | -0.10 | 1.82E-03 | -0.08 | 3.00E-39 | -0.08 | 4.31E-02 | |
| rs10048146 | 16q24.1 | 85268161 | *FOXL1* | A | 0.80 | 0.05 | 1.00E-14 | 0.12 | 3.87E-03 | 0.05 | 3.00E-11 | 0.11 | 3.27E-02 | |
| rs9921222 | 16p13.3 | 315783 | *AXIN1* | T | 0.48 | -0.04 | 5.00E-12 | -0.09 | 4.44E-03 | -0.04 | 1.00E-16 | -0.07 | 5.59E-02 | |
| rs6532023 | 4q22.1 | 88992873 | *MEPE* | T | 0.34 | 0.06 | 5.00E-26 | 0.09 | 9.63E-03 | 0.06 | 1.00E-27 | 0.09 | 2.31E-02 | |
| rs1286083 | 14q32.11 | 90512532 | *RPS6KA5* | T | 0.81 | -0.05 | 2.00E-15 | -0.10 | 1.46E-02 | -0.05 | 1.80E-14 | -0.10 | 5.63E-02 | |
| rs4792909 | 17q21.31 | 39154350 | *SOST* | T | 0.37 | 0.04 | 2.00E-11 | 0.08 | 1.53E-02 | 0.04 | 9.40E-10 | 0.00 | 9.06E-01 | |
| rs227584 | 17q21.31 | 39581073 | *C17orf53* | A | 0.70 | -0.06 | 3.00E-24 | -0.08 | 1.93E-02 | -0.04 | 9.90E-10 | -0.10 | 1.35E-02 | |
| rs6959212 | 7p14.1 | 38094851 | *STARD3NL* | T | 0.32 | -0.04 | 1.20E-13 | -0.07 | 2.34E-02 | -0.07 | 4.00E-38 | -0.09 | 3.66E-02 | |
| rs344081 | 3q25.31 | 158038678 | *LEKR1* | T | 0.87 | 0.04 | 2.22E-06 | 0.10 | 2.52E-02 | 0.06 | 4.00E-12 | 0.10 | 8.20E-02 | |
| rs2016266 | 12q13.13 | 52014222 | *SP7* | A | 0.68 | -0.03 | 3.70E-10 | -0.07 | 2.87E-02 | -0.05 | 3.00E-20 | -0.08 | 4.36E-02 | |
| rs736825 | 12q13.13 | 52703843 | *HOXC6* | C | 0.56 | 0.04 | 1.00E-09 | 0.06 | 4.68E-02 | 0.05 | 8.00E-16 | 0.06 | 1.34E-01 | |
| rs163879 | 11p14.1 | 30908250 | *DCDC5* | T | 0.68 | -0.03 | 2.00E-08 | -0.06 | 5.36E-02 | -0.04 | 2.00E-11 | -0.06 | 1.53E-01 | |
| rs7932354 | 11p11.2 | 46678797 | *ARHGAP1* | T | 0.31 | 0.05 | 5.00E-18 | 0.06 | 7.79E-02 | 0.04 | 5.40E-12 | 0.07 | 1.04E-01 | |
| rs7953528 | 12p11.22 | 27908426 | *KLHDC5* | A | 0.18 | 0.05 | 1.87E-12 | 0.07 | 9.03E-02 | -0.01 | 1.30E-01 | 0.03 | 5.92E-01 | |
| rs4790881 | 17p13.3 | 2015682 | *SMG6* | A | 0.69 | 0.05 | 1.00E-18 | 0.06 | 9.18E-02 | 0.03 | 3.40E-09 | 0.05 | 2.42E-01 | |
| rs3736228 | 11q13.2 | 67957871 | *LRP5* | T | 0.16 | -0.05 | 4.80E-11 | -0.07 | 9.54E-02 | -0.08 | 2.00E-26 | -0.13 | 1.41E-02 | |
| rs1373004 | 10q21.1 | 54097831 | *MBL2* | T | 0.13 | -0.04 | 1.45E-08 | -0.07 | 1.78E-01 | -0.06 | 2.00E-12 | -0.09 | 1.76E-01 | |
| rs17040773 | 2q13 | 112216506 | *ANAPC1* | A | 0.76 | 0.04 | 2.00E-09 | 0.05 | 2.29E-01 | 0.01 | 1.90E-01 | -0.01 | 8.42E-01 | |
| rs7217932 | 17q24.3 | 67460611 | *SOX9* | A | 0.46 | 0.03 | 2.00E-11 | 0.04 | 2.48E-01 | 0.01 | 8.00E-02 | -0.03 | 4.09E-01 | |
| rs11623869 | 14q32.32 | 102953386 | *MARK3* | T | 0.35 | -0.04 | 5.00E-16 | -0.04 | 2.69E-01 | -0.04 | 5.00E-11 | 0.01 | 8.89E-01 | |
| rs1026364 | 3q13.2 | 114852700 | *KIAA2018* | T | 0.37 | 0.03 | 4.00E-10 | 0.04 | 2.74E-01 | 0.02 | 7.57E-04 | -0.01 | 8.67E-01 | |
| rs1878526 | 2q14.2 | 118755068 | *INSIG2* | A | 0.22 | 0.00 | 7.90E-01 | 0.04 | 2.77E-01 | 0.04 | 1.00E-10 | 0.02 | 6.53E-01 | |
| rs11755164 | 6p21.1 | 44747162 | *SUPT3H* | T | 0.40 | -0.01 | 5.00E-02 | -0.03 | 3.02E-01 | -0.04 | 6.00E-11 | -0.12 | 2.29E-03 | |
| rs1564981 | 16q12.1 | 49543809 | *CYLD* | A | 0.50 | -0.02 | 4.38E-05 | -0.03 | 3.29E-01 | -0.04 | 2.00E-10 | -0.05 | 1.94E-01 | |
| rs17482952 | 1p31.3 | 68411973 | *WLS* | A | 0.07 | 0.08 | 1.31E-11 | -0.05 | 3.84E-01 | 0.07 | 1.69E-08 | 0.02 | 7.54E-01 | |
| rs3905706 | 10p12.1 | 28519948 | *MPP7* | T | 0.22 | 0.01 | 3.00E-02 | -0.03 | 4.08E-01 | 0.05 | 2.00E-16 | 0.07 | 1.45E-01 | |
| rs7084921 | 10q24.2 | 101803792 | *CPN1* | T | 0.39 | 0.03 | 9.00E-10 | 0.02 | 4.52E-01 | 0.03 | 9.00E-07 | -0.01 | 8.96E-01 | |
| rs4233949 | 2p16.2 | 54513211 | *SPTBN1* | C | 0.38 | 0.02 | 5.91E-06 | -0.02 | 6.25E-01 | 0.05 | 2.00E-18 | 0.04 | 2.37E-01 | |
| rs7017914 | 8q13.3 | 71753757 | *XKR9* | A | 0.49 | 0.03 | 2.00E-07 | 0.01 | 6.44E-01 | 0.01 | 2.60E-01 | 0.01 | 8.25E-01 | |
| rs1053051 | 12q23.3 | 105891355 | *C12orf23* | T | 0.52 | -0.03 | 1.00E-09 | -0.01 | 6.65E-01 | -0.03 | 8.00E-08 | -0.03 | 3.74E-01 | |
| rs7751941 | 6q25.1 | 151988351 | *CCDC170* | A | 0.23 | -0.04 | 1.59E-09 | -0.01 | 7.22E-01 | -0.08 | 1.99E-24 | -0.06 | 1.88E-01 | |
| rs10226308 | 7p14.1 | 37904947 | *NME8* | A | 0.84 | -0.02 | 2.00E-02 | -0.01 | 7.23E-01 | -0.06 | 6.00E-13 | -0.02 | 6.82E-01 | |
| rs7071206 | 10q22.3 | 79071322 | *KCNMA1* | T | 0.78 | 0.00 | 8.10E-01 | 0.01 | 7.46E-01 | -0.06 | 5.00E-19 | -0.12 | 1.45E-02 | |
| rs13245690 | 7q31.31 | 120572300 | *CPED1* | A | 0.65 | 0.02 | 8.20E-04 | 0.00 | 9.19E-01 | 0.05 | 2.00E-11 | 0.01 | 7.55E-01 | |
|  |  |  |  |  |  |  |  |  | | | | | |  |
| rs479336 | 1q24.3 | 170466196 | *DNM3* | T | 0.74 | -0.04 | 9.00E-15 | Concordance filtered | | | | | |  |
| rs7521902 | 1p36.12 | 22363311 | *WNT4* | A | 0.31 | -0.04 | 2.80E-09 | Not imputed | | | | | |  |
| rs3755955 | 4p16.3 | 984414 | *IDUA* | A | 0.16 | -0.06 | 1.40E-15 | Concordance filtered | | | | | |  |
| rs4869742 | 6q25.1 | 151949441 | *CCDC170* | T | 0.31 | -0.05 | 4.00E-18 | Not imputed | | | | | |  |
| rs7812088 | 7q36.1 | 150550762 | *ABCF2* | A | 0.13 | 0.05 | 7.00E-09 | Concordance filtered | | | | | |  |
| rs10835187 | 11p14.1 | 27462253 | *LIN7C* | T | 0.55 | -0.01 | 3.00E-02 | Not imputed | | | | | |  |
| rs12821008 | 12q13.12 | 47760872 | *DHH* | T | 0.39 | 0.03 | 3.30E-07 | Not in ref haps | | | | | |  |
| rs2887571 | 12p13.33 | 1508432 | *ERC1 / WNT5B* | A | 0.76 | -0.03 | 6.50E-09 | Concordance filtered | | | | | |  |
| rs4985155 | 16p13.11 | 15036960 | *NTAN1* | A | 0.67 | -0.03 | 2.00E-10 | Not imputed | | | | | |  |
| rs1566045 | 16q12.1 | 49579304 | *SALL1* | T | 0.80 | -0.06 | 2.00E-22 | InfoScore filtered | | | | | |  |
| rs13336428 | 16p13.3 | 1472464 | *PTX4* | A | 0.43 | -0.04 | 1.00E-16 | Concordance filtered | | | | | |  |
| rs1864325 | 17q21.31 | 41333623 | *MAPT / WNT3* | T | 0.22 | -0.03 | 7.47E-05 | Not in ref haps | | | | | |  |
| rs884205 | 18q21.33 | 58205837 | *TNFRSF11A* | A | 0.27 | -0.04 | 3.00E-10 | Concordance filtered | | | | | |  |
| rs3790160 | 20p12.2 | 10587988 | *JAG1* | T | 0.50 | 0.04 | 3.60E-12 | Concordance filtered | | | | | |  |
| rs5934507 | Xp22.31 | 8877206 | *FAM9B* | A | 0.73 | -0.04 | 1.64E‐04 | ChrX | | | | | |  |

HBM analysis includes HBM cases + AOGC High +AOGC Low for total hip Z-score (n=2110) and lumbar spine Z-Score (n=1380).

Adjusted for age, age2, center and population stratification

EA: Effect Allele. EAF: Effect Allele Frequency. FN: femoral neck. HIP: total hip. LS: lumbar spine. B: Beta. P: p value.

βeta: effect estimate – in HBM/AOGC represents change in BMD Z-score per copy of the SNP EA

Those rows in bold show HBM_AOGC GWAS associations with p<5x10^-05^

**Supplementary Table 3: (Stage 1: Analyses concerning established BMD-associated loci) GWAS results for 64 established BMD-associated loci published by Estrada et al, showing associations for HBM GWAS of total hip and lumbar spine, adjusting for age, age^2^, center, gender, weight and population stratification**

|  |  |  |  |  |  | **Estrada et al**  **FN** | | **HBM_AOGC**  **Total Hip** | | **Estrada et al**  **LS** | | **HBM_AOGC**  **LS** | | |
| --- | --- | --- | --- | --- | --- | --- | --- | --- | --- | --- | --- | --- | --- | --- |
| **SNPID** | **LOCUS** | **POSITION** | **GENE** | **EA** | **EAF** | **FN-B** | **FN-P** | **HIP-B** | **HIP-P** | **LS-B** | **LS-P** | **LS-B** | **LS-P** | |
| **rs1366594** | **5q14.3** | **88411817** | ***MEF2C*** | **A** | **0.54** | **0.08** | **4.00E-61** | **0.18** | **8.74E-09** | **0.01** | **1.00E-02** | **0.06** | **1.38E-01** | |
| **rs6426749** | **1p36.12** | **22584060** | ***ZBTB40/WNT4*** | **C** | **0.17** | **0.11** | **7.00E-57** | **0.19** | **2.14E-06** | **0.1** | **1.80E-44** | **0.13** | **6.77E-03** | |
| **rs7108738** | **11p15.2** | **15666660** | ***SOX6*** | **T** | **0.83** | **-0.08** | **1.00E-32** | **-0.19** | **2.28E-06** | **-0.03** | **2.14E-06** | **-0.16** | **9.30E-04** | |
| **rs430727** | **3p22.1** | **41103568** | ***CTNNB1*** | **T** | **0.48** | **-0.06** | **4.00E-25** | **-0.16** | **4.40E-07** | **-0.05** | **1.50E-18** | **-0.13** | **1.07E-03** | |
| **rs13204965** | **6q22.33** | **127208765** | ***RSPO3*** | **A** | **0.76** | **0.04** | **8.00E-12** | **0.17** | **4.45E-06** | **0.04** | **3.60E-10** | **0.12** | **6.86E-03** | |
| rs1346004 | 2q24.3 | 166309292 | *GALNT3* | A | 0.50 | -0.05 | 1.00E-25 | -0.11 | 6.57E-04 | -0.06 | 4.00E-30 | -0.05 | 1.78E-01 | |
| rs12407028 | 1p31.3 | 68420304 | *WLS* | T | 0.60 | 0.06 | 3.40E-23 | 0.10 | 2.59E-03 | 0.08 | 3.00E-45 | 0.07 | 7.06E-02 | |
| rs7851693 | 9q34.11 | 132468648 | *FUBP3* | C | 0.64 | 0.05 | 3.00E-22 | 0.11 | 8.95E-04 | 0.03 | 6.00E-08 | 0.09 | 2.24E-02 | |
| **rs4796995** | **18p11.21** | **13698574** | ***FAM210A*** | **A** | **0.63** | **0.03** | **5.00E-08** | **0.13** | **4.20E-05** | **0.02** | **6.65E-04** | **0.06** | **1.34E-01** | |
| rs7584262 | 2p21 | 42104053 | *PKDCC* | T | 0.23 | 0.04 | 1.00E-09 | 0.15 | 8.15E-05 | 0.01 | 7.00E-02 | 0.06 | 2.24E-01 | |
| rs4727338 | 7q21.3 | 95958611 | *SHFM1* | C | 0.67 | 0.08 | 8.00E-48 | 0.14 | 5.51E-05 | 0.07 | 2.00E-35 | 0.12 | 3.31E-03 | |
| rs3801387 | 7q31.31 | 120762001 | *WNT16* | A | 0.74 | -0.08 | 5.00E-40 | -0.10 | 2.26E-03 | -0.09 | 3.00E-51 | -0.05 | 2.02E-01 | |
| rs10416218 | 19q13.11 | 38290967 | *GPATCH1* | T | 0.73 | -0.03 | 5.50E-08 | -0.10 | 4.10E-03 | -0.04 | 7.00E-11 | -0.05 | 2.67E-01 | |
| rs9533090 | 13q14.11 | 41849449 | *AKAP11* | T | 0.49 | -0.05 | 5.00E-23 | -0.11 | 4.48E-04 | -0.10 | 5.00E-68 | -0.15 | 6.76E-05 | |
| rs9466056 | 6p22.3 | 21492592 | *CDKAL1* | A | 0.38 | -0.03 | 3.00E-08 | -0.10 | 1.39E-03 | -0.04 | 3.00E-13 | -0.09 | 2.75E-02 | |
| rs2062377 | 8q24.12 | 120076601 | *TNFRSF11B* | A | 0.57 | -0.06 | 9.00E-25 | -0.12 | 2.08E-04 | -0.08 | 3.00E-39 | -0.09 | 2.10E-02 | |
| rs10048146 | 16q24.1 | 85268161 | *FOXL1* | A | 0.80 | 0.05 | 1.00E-14 | 0.09 | 1.95E-02 | 0.05 | 3.00E-11 | 0.09 | 8.69E-02 | |
| rs9921222 | 16p13.3 | 315783 | *AXIN1* | T | 0.48 | -0.04 | 5.00E-12 | -0.08 | 7.98E-03 | -0.04 | 1.00E-16 | -0.05 | 1.48E-01 | |
| rs6532023 | 4q22.1 | 88992873 | *MEPE* | T | 0.34 | 0.06 | 5.00E-26 | 0.06 | 5.14E-02 | 0.06 | 1.00E-27 | 0.05 | 2.17E-01 | |
| rs1286083 | 14q32.11 | 90512532 | *RPS6KA5* | T | 0.81 | -0.05 | 2.00E-15 | -0.09 | 2.85E-02 | -0.05 | 1.80E-14 | -0.09 | 7.87E-02 | |
| rs4792909 | 17q21.31 | 39154350 | *SOST* | T | 0.37 | 0.04 | 2.00E-11 | 0.09 | 6.95E-03 | 0.04 | 9.40E-10 | 0.00 | 9.94E-01 | |
| rs227584 | 17q21.31 | 39581073 | *C17orf53* | A | 0.70 | -0.06 | 3.00E-24 | -0.08 | 2.51E-02 | -0.04 | 9.90E-10 | -0.09 | 2.85E-02 | |
| rs6959212 | 7p14.1 | 38094851 | *STARD3NL* | T | 0.32 | -0.04 | 1.20E-13 | -0.05 | 1.51E-01 | -0.07 | 4.00E-38 | -0.06 | 1.61E-01 | |
| rs344081 | 3q25.31 | 158038678 | *LEKR1* | T | 0.87 | 0.04 | 2.22E-06 | 0.09 | 4.19E-02 | 0.06 | 4.00E-12 | 0.09 | 1.20E-01 | |
| rs2016266 | 12q13.13 | 52014222 | *SP7* | A | 0.68 | -0.03 | 3.70E-10 | -0.06 | 8.48E-02 | -0.05 | 3.00E-20 | -0.05 | 2.44E-01 | |
| rs736825 | 12q13.13 | 52703843 | *HOXC6* | C | 0.56 | 0.04 | 1.00E-09 | 0.06 | 8.21E-02 | 0.05 | 8.00E-16 | 0.04 | 2.83E-01 | |
| rs163879 | 11p14.1 | 30908250 | *DCDC5* | T | 0.68 | -0.03 | 2.00E-08 | -0.05 | 1.24E-01 | -0.04 | 2.00E-11 | -0.07 | 1.03E-01 | |
| rs7932354 | 11p11.2 | 46678797 | *ARHGAP1* | T | 0.31 | 0.05 | 5.00E-18 | 0.05 | 1.40E-01 | 0.04 | 5.40E-12 | 0.07 | 8.56E-02 | |
| rs7953528 | 12p11.22 | 27908426 | *KLHDC5* | A | 0.18 | 0.05 | 1.87E-12 | 0.07 | 9.92E-02 | -0.01 | 1.30E-01 | 0.03 | 5.40E-01 | |
| rs4790881 | 17p13.3 | 2015682 | *SMG6* | A | 0.69 | 0.05 | 1.00E-18 | 0.07 | 3.12E-02 | 0.03 | 3.40E-09 | 0.05 | 2.30E-01 | |
| rs3736228 | 11q13.2 | 67957871 | *LRP5* | T | 0.16 | -0.05 | 4.80E-11 | -0.07 | 1.17E-01 | -0.08 | 2.00E-26 | -0.13 | 1.99E-02 | |
| rs1373004 | 10q21.1 | 54097831 | *MBL2* | T | 0.13 | -0.04 | 1.45E-08 | -0.15 | 4.65E-03 | -0.06 | 2.00E-12 | -0.14 | 2.74E-02 | |
| rs17040773 | 2q13 | 112216506 | *ANAPC1* | A | 0.76 | 0.04 | 2.00E-09 | 0.08 | 4.50E-02 | 0.01 | 1.90E-01 | 0.05 | 3.57E-01 | |
| rs7217932 | 17q24.3 | 67460611 | *SOX9* | A | 0.46 | 0.03 | 2.00E-11 | 0.04 | 1.87E-01 | 0.01 | 8.00E-02 | -0.02 | 6.53E-01 | |
| rs11623869 | 14q32.32 | 102953386 | *MARK3* | T | 0.35 | -0.04 | 5.00E-16 | -0.05 | 1.26E-01 | -0.04 | 5.00E-11 | 0.00 | 9.42E-01 | |
| rs1026364 | 3q13.2 | 114852700 | *KIAA2018* | T | 0.37 | 0.03 | 4.00E-10 | 0.02 | 5.93E-01 | 0.02 | 7.57E-04 | -0.03 | 5.16E-01 | |
| rs1878526 | 2q14.2 | 118755068 | *INSIG2* | A | 0.22 | 0.00 | 7.90E-01 | 0.04 | 2.79E-01 | 0.04 | 1.00E-10 | 0.02 | 6.58E-01 | |
| rs11755164 | 6p21.1 | 44747162 | *SUPT3H* | T | 0.40 | -0.01 | 5.00E-02 | -0.01 | 8.35E-01 | -0.04 | 6.00E-11 | -0.11 | 5.26E-03 | |
| rs1564981 | 16q12.1 | 49543809 | *CYLD* | A | 0.50 | -0.02 | 4.38E-05 | -0.04 | 1.68E-01 | -0.04 | 2.00E-10 | -0.05 | 2.27E-01 | |
| rs17482952 | 1p31.3 | 68411973 | *WLS* | A | 0.07 | 0.08 | 1.31E-11 | 0.01 | 8.51E-01 | 0.07 | 1.69E-08 | 0.09 | 2.24E-01 | |
| rs3905706 | 10p12.1 | 28519948 | *MPP7* | T | 0.22 | 0.01 | 3.00E-02 | -0.07 | 8.06E-02 | 0.05 | 2.00E-16 | 0.05 | 2.69E-01 | |
| rs7084921 | 10q24.2 | 101803792 | *CPN1* | T | 0.39 | 0.03 | 9.00E-10 | 0.02 | 5.10E-01 | 0.03 | 9.00E-07 | -0.01 | 7.44E-01 | |
| rs4233949 | 2p16.2 | 54513211 | *SPTBN1* | C | 0.38 | 0.02 | 5.91E-06 | -0.01 | 7.82E-01 | 0.05 | 2.00E-18 | 0.04 | 3.13E-01 | |
| rs7017914 | 8q13.3 | 71753757 | *XKR9* | A | 0.49 | 0.03 | 2.00E-07 | 0.02 | 5.44E-01 | 0.01 | 2.60E-01 | 0.01 | 7.20E-01 | |
| rs1053051 | 12q23.3 | 105891355 | *C12orf23* | T | 0.52 | -0.03 | 1.00E-09 | -0.04 | 2.46E-01 | -0.03 | 8.00E-08 | -0.05 | 2.03E-01 | |
| rs7751941 | 6q25.1 | 151988351 | *CCDC170* | A | 0.23 | -0.04 | 1.59E-09 | -0.04 | 2.91E-01 | -0.08 | 1.99E-24 | -0.08 | 7.94E-02 | |
| rs10226308 | 7p14.1 | 37904947 | *NME8* | A | 0.84 | -0.02 | 2.00E-02 | -0.01 | 7.72E-01 | -0.06 | 6.00E-13 | -0.03 | 5.78E-01 | |
| rs7071206 | 10q22.3 | 79071322 | *KCNMA1* | T | 0.78 | 0.00 | 8.10E-01 | 0.02 | 6.06E-01 | -0.06 | 5.00E-19 | -0.12 | 1.56E-02 | |
| rs13245690 | 7q31.31 | 120572300 | *CPED1* | A | 0.65 | 0.02 | 8.20E-04 | 0.00 | 9.09E-01 | 0.05 | 2.00E-11 | 0.01 | 7.52E-01 | |
|  |  |  |  |  |  |  |  |  | | | | | |  |
| rs479336 | 1q24.3 | 170466196 | *DNM3* | T | 0.74 | -0.04 | 9.00E-15 | Concordance filtered | | | | | |  |
| rs7521902 | 1p36.12 | 22363311 | *WNT4* | A | 0.31 | -0.04 | 2.80E-09 | Not imputed | | | | | |  |
| rs3755955 | 4p16.3 | 984414 | *IDUA* | A | 0.16 | -0.06 | 1.40E-15 | Concordance filtered | | | | | |  |
| rs4869742 | 6q25.1 | 151949441 | *CCDC170* | T | 0.31 | -0.05 | 4.00E-18 | Not imputed | | | | | |  |
| rs7812088 | 7q36.1 | 150550762 | *ABCF2* | A | 0.13 | 0.05 | 7.00E-09 | Concordance filtered | | | | | |  |
| rs10835187 | 11p14.1 | 27462253 | *LIN7C* | T | 0.55 | -0.01 | 3.00E-02 | Not imputed | | | | | |  |
| rs12821008 | 12q13.12 | 47760872 | *DHH* | T | 0.39 | 0.03 | 3.30E-07 | Not in ref haps | | | | | |  |
| rs2887571 | 12p13.33 | 1508432 | *ERC1 / WNT5B* | A | 0.76 | -0.03 | 6.50E-09 | Concordance filtered | | | | | |  |
| rs4985155 | 16p13.11 | 15036960 | *NTAN1* | A | 0.67 | -0.03 | 2.00E-10 | Not imputed | | | | | |  |
| rs1566045 | 16q12.1 | 49579304 | *SALL1* | T | 0.80 | -0.06 | 2.00E-22 | InfoScore filtered | | | | | |  |
| rs13336428 | 16p13.3 | 1472464 | *PTX4* | A | 0.43 | -0.04 | 1.00E-16 | Concordance filtered | | | | | |  |
| rs1864325 | 17q21.31 | 41333623 | *MAPT / WNT3* | T | 0.22 | -0.03 | 7.47E-05 | Not in ref haps | | | | | |  |
| rs884205 | 18q21.33 | 58205837 | *TNFRSF11A* | A | 0.27 | -0.04 | 3.00E-10 | Concordance filtered | | | | | |  |
| rs3790160 | 20p12.2 | 10587988 | *JAG1* | T | 0.50 | 0.04 | 3.60E-12 | Concordance filtered | | | | | |  |
| rs5934507 | Xp22.31 | 8877206 | *FAM9B* | A | 0.73 | -0.04 | 1.64E‐04 | ChrX | | | | | |  |

HBM analysis includes HBM cases + AOGC High +AOGC Low for total hip Z-score (n=2110) and lumbar spine Z-Score (n=1380).

Adjusted for age, age2, center, gender, weight and population stratification

EA: Effect Allele. EAF: Effect Allele Frequency. FN: femoral neck. HIP: total hip. LS: lumbar spine. B: Beta. P: p value.

βeta: effect estimate – in HBM represents change in BMD Z-score per copy of the SNP EA; βeta: effect estimate – in GEFOS represents change in standard deviations of BMD per copy of the SNP EA

Those rows in bold show HBM_AOGC GWAS associations with p<5x10^-05^

**Supplementary Table 4: (Stage 1: GWAS discovery) Sensitivity Analysis:** **Genome-wide significant SNPs with p<5x10^-8^ associated with BMD Z-score measured at the total hip and lumbar spine after additional height adjustment**

| **rsID** | **Locus** | **Position** | **Closest gene / candidate** | **EA** | **EAF** | **Total Hip BMD Z-score** | | | | **Lumbar Spine BMD Z-score** | | | |
| --- | --- | --- | --- | --- | --- | --- | --- | --- | --- | --- | --- | --- | --- |
|  |  |  |  |  |  | **n** | **β** | **SE** | **p** | **n** | **β** | **SE** | **p** |
| rs1366594 | 5q14.3 | 88376061 | *MEF2C* | C | 0.47 | 2094 | -0.180 | 0.031 | **5.58 x10^-09^** | 1380 | -0.091 | 0.039 | 1.79 x10^-02^ |
| rs113784679 | 1p36.12 | 22648479 | *WNT4/*  *ZBTB40* | T | 0.04 | 2094 | 0.515 | 0.089 | **8.97 x10^-09^** | 1380 | 0.221 | 0.115 | 5.47 x10^-02^ |
| rs9292469 | 5p13.3 | 32840210 | *NPR3* | T | 0.33 | 2094 | 0.111 | 0.033 | 8.16 x10^-04^ | 1380 | 0.233 | 0.041 | **2.25 x10^-08^** |
| rs2697825 | 11p15.2 | 14089431 | *SPON1* | G | 0.17 | 1078^a^ | 0.250 | 0.057 | 1.16 x10^-05^ | 767^a^ | 0.148 | 0.065 | 2.30 x10^-02^ |

Chromosome position using: GRCh37.p13

Quantitative analysis results shown use data from HBM, AOGC high BMD and AOGC low BMD cohorts, except ^a^ which reflects analyses restricted to HBM and AOGC low BMD cohorts. Model is adjusted for age, age^2^, center, 4 PCs and HEIGHT

p < 5 x 10^-8^ appear in bold

EA: Effect Allele (BMD increasing); EAF: Effect Allele Frequency. SE: Standard Error. β: effect estimate – represents change in BMD Z-score per copy of the SNP EA

**Supplementary Table 5: (Stage 1: GWAS discovery) 75 Suggestive SNPs with p<5x10-5 associated with BMD Z-score measured at the total hip in Stage 1 GWAS**

| **SNP** | **CHR** | **POS** | **Closest gene(s) / candidate(s)** | **Region** | **EA** | **EAF** | **BETA** | **SE** | **P VALUE** | **SNP TYPE** | **Analysis** |
| --- | --- | --- | --- | --- | --- | --- | --- | --- | --- | --- | --- |
| rs416486 | 3 | 41113076 | *ZNF621,CTNNB1* | intergenic | T | 0.44 | -0.157 | 0.031 | 3.95E-07 | Imputed | 1 |
| rs1531903 | 11 | 15668826 | *LOC102724957* | intronic | C | 0.18 | 0.203 | 0.040 | 5.36E-07 | Imputed | 1 |
| rs10860802 | 12 | 102265606 | *GNPTAB,DRAM1* | intergenic | G | 0.17 | -0.199 | 0.042 | 2.67E-06 | Imputed | 1 |
| rs3815993 | 2 | 166732337 | *TTC21B* | intronic | A | 0.18 | -0.195 | 0.042 | 3.89E-06 | Imputed | 1 |
| rs28669331 | 20 | 19909821 | *RIN2* | intronic | C | 0.03 | -0.452 | 0.100 | 6.03E-06 | Imputed | 1 |
| rs11692416 | 2 | 36710779 | *CRIM1* | intronic | T | 0.37 | 0.146 | 0.033 | 7.91E-06 | Imputed | 1 |
| rs7823971 | 8 | 72471728 | *EYA1,MSC* | intergenic | A | 0.20 | -0.167 | 0.038 | 1.11E-05 | Imputed | 1 |
| rs10929283 | 2 | 239489310 | *LINC01107,TWIST2* | intergenic | A | 0.48 | 0.135 | 0.031 | 1.31E-05 | Imputed | 1 |
| rs75875965 | 13 | 42974206 | *AKAP11,TNFSF11* | intergenic | T | 0.05 | 0.296 | 0.068 | 1.45E-05 | Imputed | 1 |
| rs236956 | 6 | 16570661 | *ATXN1* | intronic | A | 0.41 | -0.134 | 0.031 | 1.85E-05 | Imputed | 1 |
| rs841037 | 10 | 125366785 | *BUB3,GPR26* | intergenic | C | 0.03 | 0.405 | 0.094 | 1.86E-05 | Imputed | 1 |
| rs73063011 | 19 | 52338183 | *FPR3,ZNF577* | intergenic | T | 0.10 | -0.227 | 0.053 | 2.14E-05 | Imputed | 1 |
| rs1043925 | 18 | 13764016 | *RNMT* | UTR3 | A | 0.49 | 0.128 | 0.030 | 2.23E-05 | Imputed | 1 |
| rs141673734 | 1 | 182432199 | *RGSL1* | intronic | T | 0.08 | 0.252 | 0.059 | 2.3E-05 | Imputed | 1 |
| rs9929301 | 16 | 86521590 | *FENDRR* | ncRNA_exonic | C | 0.10 | -0.222 | 0.052 | 2.33E-05 | Imputed | 1 |
| rs10263404 | 7 | 32218987 | *PDE1C* | intronic | T | 0.32 | -0.139 | 0.033 | 2.38E-05 | Imputed | 1 |
| rs7646649 | 3 | 185690726 | *LOC344887* | ncRNA_intronic | T | 0.26 | 0.153 | 0.036 | 2.39E-05 | Imputed | 1 |
| rs143312401 | 8 | 76321156 | *CASC9,HNF4G* | intergenic | T | 0.03 | -0.377 | 0.090 | 2.7E-05 | Imputed | 1 |
| rs55738160 | 10 | 89318235 | *MINPP1,PAPSS2* | intergenic | G | 0.04 | -0.341 | 0.081 | 2.97E-05 | Imputed | 1 |
| rs1330883 | 13 | 78658633 | *RNF219-AS1* | ncRNA_intronic | T | 0.39 | 0.134 | 0.032 | 2.98E-05 | Imputed | 1 |
| rs11607554 | 11 | 86376341 | *ME3* | intronic | A | 0.42 | 0.131 | 0.032 | 3.34E-05 | Imputed | 1 |
| rs5767881 | 22 | 48045889 | *LOC284930* | ncRNA_intronic | G | 0.32 | 0.140 | 0.034 | 3.45E-05 | Genotyped | 1 |
| rs9362639 | 6 | 90055445 | *UBE2J1* | intronic | A | 0.50 | -0.129 | 0.031 | 3.53E-05 | Imputed | 1 |
| 15-50724773 | 15 | 50724773 | *USP8* | intronic | G | 0.05 | 0.299 | 0.072 | 3.64E-05 | Imputed | 1 |
| rs4722127 | 7 | 22363812 | *RAPGEF5* | intronic | C | 0.06 | -0.267 | 0.065 | 3.69E-05 | Imputed | 1 |
| rs1019000 | 7 | 50809771 | *GRB10* | intronic | T | 0.28 | 0.142 | 0.034 | 3.7E-05 | Imputed | 1 |
| rs4588689 | 6 | 12955611 | *PHACTR1* | intronic | T | 0.09 | 0.236 | 0.058 | 4.42E-05 | Imputed | 1 |
| rs12536721 | 7 | 53247212 | *POM121L12,LINC01446* | intergenic | C | 0.22 | 0.151 | 0.037 | 4.54E-05 | Imputed | 1 |
| rs143652203 | 3 | 120256419 | *FSTL1,NDUFB4* | intergenic | T | 0.14 | -0.179 | 0.044 | 4.73E-05 | Imputed | 1 |
| rs246829 | 7 | 12982439 | *ARL4A,ETV1* | intergenic | T | 0.39 | 0.129 | 0.032 | 4.87E-05 | Genotyped | 1 |
| rs2469956 | 2 | 204375993 | *RAPH1* | intronic | T | 0.04 | 0.630 | 0.121 | 2.41E-07 | Imputed | 2 |
| rs1214208 | 12 | 128282212 | *LOC101927637,FLJ37505* | intergenic | G | 0.09 | -0.397 | 0.080 | 8.56E-07 | Imputed | 2 |
| rs2226992 | 16 | 13650148 | *SHISA9,ERCC4* | intergenic | T | 0.28 | -0.241 | 0.049 | 1.23E-06 | Imputed | 2 |
| rs12322049 | 12 | 114786218 | *RBM19,TBX5* | intergenic | A | 0.07 | 0.406 | 0.085 | 2.08E-06 | Genotyped | 2 |
| rs113022464 | 2 | 27633646 | *PPM1G,NRBP1* | intergenic | G | 0.43 | 0.218 | 0.048 | 5.29E-06 | Imputed | 2 |
| rs6822446 | 4 | 129286603 | *PGRMC2,JADE1* | intergenic | A | 0.18 | 0.259 | 0.057 | 5.97E-06 | Imputed | 2 |
| rs8080236 | 17 | 69342363 | *CASC17,LOC102723505* | intergenic | C | 0.35 | 0.207 | 0.046 | 6.05E-06 | Imputed | 2 |
| rs12272967 | 11 | 15630635 | *INSC,LOC102724957* | intergenic | A | 0.42 | 0.206 | 0.045 | 6.39E-06 | Imputed | 2 |
| rs28608951 | 21 | 46900546 | *COL18A1* | intronic | T | 0.29 | 0.216 | 0.048 | 8.16E-06 | Imputed | 2 |
| rs12610303 | 19 | 28904814 | *LOC101927151,LOC100420587* | intergenic | C | 0.29 | -0.228 | 0.051 | 8.27E-06 | Imputed | 2 |
| rs10119402 | 9 | 111938125 | *EPB41L4B* | intronic | G | 0.21 | 0.231 | 0.052 | 1.1E-05 | Genotyped | 2 |
| rs6713422 | 2 | 79100366 | *LOC101927967,REG3G* | intergenic | G | 0.28 | 0.207 | 0.047 | 1.2E-05 | Imputed | 2 |
| rs7317951 | 13 | 77991150 | *MYCBP2,SCEL* | intergenic | A | 0.32 | 0.200 | 0.045 | 1.26E-05 | Imputed | 2 |
| rs2826180 | 21 | 21702869 | *LOC101927797,LINC00320* | intergenic | G | 0.39 | 0.197 | 0.045 | 1.36E-05 | Genotyped | 2 |
| rs12786819 | 11 | 19994529 | *NAV2* | intronic | T | 0.16 | 0.261 | 0.060 | 1.39E-05 | Genotyped | 2 |
| rs57122026 | 7 | 129174189 | *SMKR1,NRF1* | intergenic | A | 0.31 | 0.219 | 0.050 | 1.49E-05 | Imputed | 2 |
| rs62273061 | 3 | 134711928 | *EPHB1* | intronic | A | 0.15 | 0.261 | 0.060 | 1.53E-05 | Imputed | 2 |
| rs75499190 | 1 | 212691289 | *NENF,ATF3* | intergenic | C | 0.21 | 0.237 | 0.055 | 1.82E-05 | Imputed | 2 |
| rs1277617 | 14 | 30609489 | *PRKD1,G2E3* | intergenic | T | 0.08 | 0.359 | 0.084 | 2.13E-05 | Imputed | 2 |
| rs146407856 | 8 | 127353935 | *LINC00861,FAM84B* | intergenic | A | 0.01 | 0.867 | 0.203 | 2.13E-05 | Imputed | 2 |
| rs12440410 | 15 | 58433686 | *AQP9* | intronic | C | 0.04 | -0.469 | 0.110 | 2.18E-05 | Genotyped | 2 |
| rs10740210 | 10 | 67478463 | *LOC101928913* | ncRNA_intronic | T | 0.10 | 0.308 | 0.072 | 2.25E-05 | Genotyped | 2 |
| rs16950625 | 15 | 67417670 | *SMAD3* | intronic | A | 0.08 | 0.350 | 0.082 | 2.28E-05 | Imputed | 2 |
| rs79113690 | 7 | 101962374 | *SH2B2* | downstream | A | 0.06 | 0.413 | 0.097 | 2.34E-05 | Imputed | 2 |
| rs111923076 | 20 | 34271380 | *NFS1* | intronic | C | 0.04 | 0.477 | 0.112 | 2.35E-05 | Imputed | 2 |
| rs2134357 | 3 | 2472932 | *CNTN4* | intronic | G | 0.32 | -0.194 | 0.046 | 2.39E-05 | Genotyped | 2 |
| rs56048557 | 7 | 46016345 | *IGFBP3,TNS3* | intergenic | C | 0.16 | 0.262 | 0.062 | 2.4E-05 | Imputed | 2 |
| rs7630537 | 3 | 45131018 | *CDCP1* | intronic | A | 0.28 | -0.219 | 0.052 | 2.44E-05 | Imputed | 2 |
| rs12448573 | 16 | 3444157 | *ZSCAN32* | intronic | T | 0.47 | 0.181 | 0.043 | 2.46E-05 | Imputed | 2 |
| rs112462588 | 4 | 118333836 | *TRAM1L1,NDST3* | intergenic | T | 0.04 | 0.457 | 0.108 | 2.47E-05 | Imputed | 2 |
| rs7144349 | 14 | 21438955 | *RNASE2,METTL17* | intergenic | T | 0.37 | 0.192 | 0.046 | 2.67E-05 | Imputed | 2 |
| rs7977973 | 12 | 40927890 | *MUC19* | intronic | G | 0.34 | -0.185 | 0.044 | 2.86E-05 | Genotyped | 2 |
| rs139392063 | 5 | 60134638 | *ELOVL7* | intronic | A | 0.07 | -0.351 | 0.084 | 2.94E-05 | Imputed | 2 |
| rs73787170 | 5 | 110759964 | *CAMK4* | intronic | G | 0.33 | -0.193 | 0.046 | 2.99E-05 | Imputed | 2 |
| rs1530994 | 16 | 16964436 | *NPIPA7,XYLT1* | intergenic | A | 0.38 | -0.187 | 0.045 | 3.21E-05 | Imputed | 2 |
| rs10786776 | 10 | 105662994 | *OBFC1* | intronic | G | 0.40 | -0.182 | 0.044 | 3.58E-05 | Imputed | 2 |
| rs13180157 | 5 | 54232372 | *LOC102467080,ESM1* | intergenic | A | 0.38 | -0.189 | 0.046 | 3.79E-05 | Genotyped | 2 |
| rs2422940 | 20 | 4725377 | *PRNT,RASSF2* | intergenic | C | 0.04 | 0.466 | 0.113 | 4.02E-05 | Imputed | 2 |
| rs6443644 | 3 | 179097134 | *MFN1* | intronic | T | 0.30 | -0.210 | 0.051 | 4.08E-05 | Imputed | 2 |
| rs111808561 | 8 | 11682858 | *FDFT1* | intronic | C | 0.16 | -0.249 | 0.060 | 4.1E-05 | Imputed | 2 |
| rs1876437 | 17 | 27536374 | *MYO18A,CRYBA1* | intergenic | T | 0.24 | 0.214 | 0.052 | 4.1E-05 | Genotyped | 2 |
| rs12443320 | 15 | 50234263 | *ATP8B4* | intronic | A | 0.04 | -0.451 | 0.110 | 4.17E-05 | Genotyped | 2 |
| rs926204 | 21 | 36592062 | *RUNX1,LOC100506403* | intergenic | G | 0.02 | 0.687 | 0.168 | 4.43E-05 | Genotyped | 2 |
| rs112382203 | 4 | 189443134 | *LINC01060* | ncRNA_intronic | T | 0.14 | 0.262 | 0.064 | 4.61E-05 | Imputed | 2 |
| rs12952985 | 17 | 1692974 | *SMYD4* | intronic | G | 0.19 | -0.236 | 0.058 | 4.69E-05 | Imputed | 2 |

Chromosome position using: GRCh37.p13

NB. Where SNP associations with p<5x10-5 were found in both analysis 1 and 2, the most significant association is shown

Analysis (1) includes HBM cases+AOGC High +AOGC Low for total hip Z-Score (n=2110)

Analysis (2) includes HBM cases+AOGC Low for total hip Z-Score (n=1084)

βeta: effect estimate – represents change in BMD Z-score per copy of the SNP EA

**Supplementary Table 6: (Stage 1: GWAS discovery) 71 Suggestive SNPs with p<5x10-5 associated with BMD Z-score measured at the lumbar spine in Stage 1 GWAS**

| **SNP** | **CHR** | **POS** | **Closest gene(s) / candidate(s)** | **Region** | **EA** | **EAF** | **BETA** | **SE** | **P VALUE** | **SNP TYPE** | **Analysis** |
| --- | --- | --- | --- | --- | --- | --- | --- | --- | --- | --- | --- |
| rs7234864 | 18 | 57734857 | *PMAIP1,MC4R* | intergenic | T | 0.28 | 0.287 | 0.056 | 3.081E-07 | Genotyped | 2 |
| rs2834062 | 21 | 34377485 | *C21orf62,OLIG2* | intergenic | A | 0.37 | 0.290 | 0.056 | 3.217E-07 | Genotyped | 2 |
| rs1975031 | 19 | 7217601 | *INSR* | intronic | A | 0.22 | 0.324 | 0.064 | 5.113E-07 | Imputed | 2 |
| rs6822446 | 4 | 129286603 | *PGRMC2,JADE1* | intergenic | A | 0.18 | 0.321 | 0.065 | 9.51E-07 | Imputed | 2 |
| rs73898803 | 20 | 10631561 | *JAG1* | intronic | T | 0.07 | -0.360 | 0.075 | 1.891E-06 | Imputed | 1 |
| rs2315489 | 3 | 69511431 | *FRMD4B,MITF* | intergenic | A | 0.33 | -0.272 | 0.057 | 1.93E-06 | Imputed | 2 |
| rs7404312 | 16 | 78849375 | *WWOX* | intronic | T | 0.49 | 0.239 | 0.050 | 2.038E-06 | Genotyped | 2 |
| rs72932079 | 6 | 94768037 | *TSG1,MANEA-AS1* | intergenic | G | 0.01 | 1.124 | 0.236 | 2.275E-06 | Imputed | 2 |
| rs141698759 | 4 | 18600084 | *LCORL,SLIT2* | intergenic | T | 0.17 | -0.314 | 0.068 | 4.85E-06 | Imputed | 2 |
| rs34407399 | 4 | 178260485 | *NEIL3* | intronic | A | 0.06 | 0.378 | 0.083 | 5.872E-06 | Imputed | 1 |
| rs11589130 | 1 | 233074178 | *MAP10,NTPCR* | intergenic | C | 0.17 | -0.316 | 0.069 | 6.326E-06 | Imputed | 2 |
| rs8030719 | 15 | 35136247 | *LOC101928174,AQR* | intergenic | A | 0.16 | -0.233 | 0.052 | 7.289E-06 | Imputed | 1 |
| rs66618019 | 3 | 115143807 | *ZBTB20,GAP43* | intergenic | T | 0.09 | 0.389 | 0.087 | 8.255E-06 | Imputed | 2 |
| rs55778791 | 12 | 104915907 | *CHST11* | intronic | T | 0.34 | 0.179 | 0.040 | 8.523E-06 | Imputed | 1 |
| rs12441380 | 15 | 94864581 | *MCTP2* | intronic | A | 0.20 | -0.298 | 0.067 | 9.244E-06 | Imputed | 2 |
| rs9530838 | 13 | 79519648 | *LINC00331,RBM26* | intergenic | C | 0.36 | 0.232 | 0.052 | 9.28E-06 | Imputed | 2 |
| rs7128738 | 11 | 15692228 | *LOC102724957* | intronic | T | 0.22 | 0.204 | 0.046 | 9.885E-06 | Genotyped | 1 |
| rs61934755 | 12 | 63810195 | *AVPR1A,DPY19L2* | intergenic | T | 0.18 | -0.219 | 0.049 | 1.03E-05 | Imputed | 1 |
| rs10893007 | 11 | 123184109 | *CLMP,MIR4493* | intergenic | C | 0.02 | -0.630 | 0.142 | 1.046E-05 | Imputed | 1 |
| rs11171980 | 12 | 57200181 | *HSD17B6,SDR9C7* | intergenic | A | 0.29 | -0.183 | 0.042 | 1.108E-05 | Imputed | 1 |
| rs4841254 | 8 | 9791143 | *MIR124-1,MSRA* | intergenic | G | 0.35 | 0.239 | 0.054 | 1.108E-05 | Genotyped | 2 |
| rs17034560 | 1 | 10229158 | *UBE4B* | intronic | A | 0.04 | 0.454 | 0.103 | 1.113E-05 | Imputed | 1 |
| rs115309329 | 1 | 90844019 | *ZNF326,BARHL2* | intergenic | G | 0.06 | 0.502 | 0.114 | 1.13E-05 | Imputed | 2 |
| rs7351030 | 18 | 71270028 | *LOC100505817,FBXO15* | intergenic | A | 0.37 | 0.173 | 0.039 | 1.197E-05 | Imputed | 1 |
| rs114749882 | 1 | 9987135 | *CTNNBIP1,LZIC* | intergenic | A | 0.02 | 0.564 | 0.129 | 1.253E-05 | Imputed | 1 |
| 9-110430408 | 9 | 110430408 | *KLF4,ACTL7B* | intergenic | A | 0.37 | 0.245 | 0.056 | 1.258E-05 | Imputed | 2 |
| rs80088183 | 7 | 37751846 | *ELMO1,GPR141* | intergenic | C | 0.18 | -0.298 | 0.068 | 1.312E-05 | Imputed | 2 |
| rs7771196 | 6 | 40124947 | *MOCS1,LINC00951* | intergenic | T | 0.46 | 0.227 | 0.052 | 1.328E-05 | Imputed | 2 |
| rs79870297 | 4 | 186614953 | *SORBS2* | intronic | T | 0.23 | -0.271 | 0.062 | 1.404E-05 | Imputed | 2 |
| rs7600540 | 2 | 54993808 | *EML6* | intronic | A | 0.26 | -0.185 | 0.043 | 1.532E-05 | Imputed | 1 |
| rs12467450 | 2 | 161649113 | *RBMS1,TANK* | intergenic | G | 0.07 | 0.318 | 0.073 | 1.563E-05 | Imputed | 1 |
| rs17693567 | 8 | 87813527 | *CNGB3,CNBD1* | intergenic | G | 0.28 | -0.239 | 0.055 | 1.575E-05 | Genotyped | 2 |
| rs74922366 | 16 | 27594206 | *KIAA0556* | intronic | A | 0.05 | 0.555 | 0.128 | 1.6E-05 | Imputed | 2 |
| rs62566517 | 9 | 107761822 | *ABCA1,SLC44A1* | intergenic | A | 0.10 | 0.357 | 0.082 | 1.605E-05 | Imputed | 2 |
| rs79070173 | 3 | 20333812 | *SGOL1-AS1,LOC101927829* | intergenic | A | 0.04 | 0.459 | 0.106 | 1.609E-05 | Imputed | 1 |
| rs17105982 | 14 | 37493379 | *SLC25A21* | intronic | C | 0.10 | 0.272 | 0.063 | 1.89E-05 | Imputed | 1 |
| rs7001296 | 8 | 12822136 | *KIAA1456* | intronic | C | 0.04 | 0.579 | 0.135 | 1.925E-05 | Imputed | 2 |
| rs28843704 | 13 | 95972560 | *ABCC4,CLDN10* | intergenic | T | 0.03 | 0.519 | 0.121 | 1.984E-05 | Imputed | 1 |
| rs775194 | 11 | 109440253 | *C11orf87,ZC3H12C* | intergenic | T | 0.37 | -0.234 | 0.055 | 2.102E-05 | Imputed | 2 |
| rs10089866 | 8 | 106734859 | *ZFPM2* | intronic | T | 0.31 | 0.240 | 0.056 | 2.155E-05 | Imputed | 2 |
| rs72768619 | 16 | 24682117 | *LOC400511* | ncRNA_intronic | T | 0.07 | 0.355 | 0.083 | 2.173E-05 | Imputed | 1 |
| rs60488332 | 2 | 232425953 | *NMUR1,C2orf57* | intergenic | T | 0.44 | 0.162 | 0.038 | 2.539E-05 | Imputed | 1 |
| rs9998733 | 4 | 86789528 | *ARHGAP24* | intronic | G | 0.31 | 0.177 | 0.042 | 2.735E-05 | Imputed | 1 |
| rs13028626 | 2 | 145824195 | *TEX41* | ncRNA_intronic | A | 0.38 | -0.222 | 0.053 | 2.811E-05 | Imputed | 2 |
| rs6984049 | 8 | 11676682 | *FDFT1* | intronic | A | 0.16 | -0.298 | 0.071 | 2.847E-05 | Imputed | 2 |
| rs111381710 | 13 | 93748767 | *GPC5,GPC6* | intergenic | A | 0.50 | 0.174 | 0.041 | 2.873E-05 | Imputed | 1 |
| rs3771869 | 2 | 75424900 | *TACR1* | intronic | T | 0.45 | 0.217 | 0.052 | 2.995E-05 | Imputed | 2 |
| rs115243119 | 4 | 28733533 | *STIM2,MIR4275* | intergenic | T | 0.04 | 0.423 | 0.101 | 3.055E-05 | Imputed | 1 |
| rs36019481 | 3 | 74103399 | *PDZRN3-AS1,CNTN3* | intergenic | T | 0.23 | -0.260 | 0.062 | 3.109E-05 | Imputed | 2 |
| rs117713180 | 13 | 58853599 | *LOC101926897,DIAPH3* | intergenic | A | 0.13 | -0.250 | 0.060 | 3.115E-05 | Imputed | 1 |
| rs16938913 | 8 | 75415565 | *GDAP1,MIR5681A* | intergenic | T | 0.05 | 0.370 | 0.088 | 3.141E-05 | Imputed | 1 |
| rs56376599 | 18 | 73720917 | *SMIM21,LOC339298* | intergenic | T | 0.07 | 0.325 | 0.078 | 3.187E-05 | Imputed | 1 |
| rs11218058 | 11 | 98888865 | *MIR7976,CNTN5* | intergenic | C | 0.43 | 0.160 | 0.038 | 3.21E-05 | Imputed | 1 |
| rs321977 | 7 | 78310853 | *MAGI2* | intronic | G | 0.15 | -0.220 | 0.053 | 3.298E-05 | Imputed | 1 |
| rs7734036 | 5 | 16811774 | *MYO10* | intronic | T | 0.16 | 0.217 | 0.052 | 3.302E-05 | Imputed | 1 |
| rs7955460 | 12 | 129135840 | *TMEM132C* | intronic | T | 0.19 | -0.290 | 0.069 | 3.357E-05 | Imputed | 2 |
| rs10796101 | 10 | 13657732 | *PRPF18* | intronic | A | 0.47 | -0.214 | 0.051 | 3.451E-05 | Imputed | 2 |
| rs112714459 | 8 | 4382683 | *CSMD1* | intronic | G | 0.08 | 0.291 | 0.070 | 3.673E-05 | Imputed | 1 |
| rs74911469 | 16 | 8449573 | *RBFOX1,TMEM114* | intergenic | A | 0.02 | 0.738 | 0.178 | 3.681E-05 | Imputed | 2 |
| rs7155631 | 14 | 49115994 | *LINC00648,RPS29* | intergenic | A | 0.35 | 0.164 | 0.040 | 3.948E-05 | Imputed | 1 |
| rs35054354 | 11 | 28644443 | *MIR8068,KCNA4* | intergenic | A | 0.35 | 0.220 | 0.053 | 4.029E-05 | Imputed | 2 |
| rs35035907 | 5 | 17283505 | *BASP1,LOC401177* | intergenic | T | 0.33 | -0.239 | 0.058 | 4.069E-05 | Imputed | 2 |
| rs7842753 | 8 | 136605284 | *KHDRBS3* | intronic | G | 0.26 | -0.243 | 0.059 | 4.102E-05 | Imputed | 2 |
| rs11639601 | 16 | 7686528 | *RBFOX1* | intronic | C | 0.43 | 0.165 | 0.040 | 4.498E-05 | Imputed | 1 |
| rs141316188 | 13 | 70607534 | *KLHL1* | intronic | C | 0.12 | 0.330 | 0.080 | 4.508E-05 | Imputed | 2 |
| rs16939142 | 8 | 76625797 | *HNF4G,LINC01111* | intergenic | G | 0.02 | 0.648 | 0.158 | 4.537E-05 | Imputed | 1 |
| rs7107604 | 11 | 133389853 | *OPCML* | intronic | T | 0.04 | 0.542 | 0.132 | 4.583E-05 | Imputed | 2 |
| rs6796601 | 3 | 182648143 | *ATP11B,DCUN1D1* | intergenic | G | 0.34 | -0.168 | 0.041 | 4.595E-05 | Imputed | 1 |
| rs4976587 | 5 | 167681861 | *TENM2* | intronic | C | 0.40 | -0.220 | 0.054 | 4.642E-05 | Imputed | 2 |
| rs2270082 | 19 | 3643678 | *PIP5K1C* | intronic | A | 0.08 | 0.399 | 0.097 | 4.652E-05 | Imputed | 2 |
| rs57002554 | 10 | 27605004 | *LRRC37A6P,PTCHD3* | intergenic | G | 0.21 | -0.271 | 0.066 | 4.796E-05 | Imputed | 2 |

Chromosome position using: GRCh37.p13

NB. Where SNP associations with p<5x10-5 were found in both analysis 1 and 2, the most significant association is shown

Analysis (1) includes HBM cases+AOGC High +AOGC Low for lumbar spine Z-Score (n=1380)

Analysis (2) includes HBM cases+AOGC Low for lumbar spine Z-Score (n=767)

βeta: effect estimate – represents change in BMD Z-score per copy of the SNP EA

**Supplementary Table 7: (Stage 3: Gene expression) Results from *cis*-eQTL analyses in primary osteoblast samples taken from 95 human donors for four lead SNPs which reached GWAS significance**

|  |  |  |  |  | **HBM_AOGC GWAS Results** | | | **Osteoblast *cis*-eQTL Results** | | | | |
| --- | --- | --- | --- | --- | --- | --- | --- | --- | --- | --- | --- | --- |
| **rsID** | **Chr** | **Position** | **EA** | **EAF** | **Beta** | **SE** | **p value** | **Beta** | **SE** | **p value** | **Probe ID** | **Probe gene** |
| rs1366594 | 5 | 88376061 | C | 0.47 | -0.191 | 0.031 | 4.83 x10^-10^ | 0.038 | 0.054 | 0.485 | ILMN 19661 | *MEF2C* |
| rs113784679 | 1 | 22648479 | T | 0.04 | 0.511 | 0.088 | 8.40 x10^-09^ | -0.030 | 0.009 | 0.002 | ILMN 22408 | *WNT4* |
| rs9292469 | 5 | 32840210 | T | 0.33 | 0.232 | 0.041 | 2.70 x10^-08^ | -0.0416 | 0.016 | 0.013 | ILMN 30098 | *MTMR12* |
| rs2697825 | 11 | 14089431 | G | 0.17 | 0.310 ^a^ | 0.056 | 4.93 x10^-08^ | -0.0635 | 0.025 | 0.015 | ILMN 30066 | *BTBD10* |

Chromosome position using: GRCh37.p13

Quantitative analysis results shown use data from HBM, AOGC high BMD and AOGC low BMD cohorts, except ^a^ which reflects analyses restricted to HBM and AOGC low BMD cohorts. Model is adjusted for age, age^2^, center and 4 PCs

EA: Effect Allele; EAF: Effect Allele Frequency. SE: Standard Error.

**Supplementary Table 8: (Stage 3: Gene expression) Results from *cis*-eQTL analyses in primary osteoblasts for rs9292469** **and SNPs in Linkage Disequilibrium (r^2^>0.8)**

|  |  |  |  |  | **HBM_AOGC GWAS Results** | | | **Osteoblast *cis*-eQTL Results** | | | |
| --- | --- | --- | --- | --- | --- | --- | --- | --- | --- | --- | --- |
| **rsID** | **Chr** | **Position** | **EA** | **EAF** | **Beta** | **SE** | **p value** | **Beta** | **SE** | **p value** | **Probe Gene** |
| rs13159981 | 5 | 32829055 | T | 0.21 | 0.199 | 0.048 | 3.26E-05 | -0.0343 | 0.018 | 0.061 | *MTMR12* |
| rs1173723 | 5 | 32835212 | G | 0.48 | 0.176 | 0.039 | 5.90E-06 | -0.0542 | 0.028 | 0.056 | *SUB1* |
| rs1173720 | 5 | 32835752 | C | 0.47 | 0.175 | 0.039 | 8.50E-06 | 0.0825 | 0.043 | 0.055 | *TARS* |
| rs10066652 | 5 | 32835753 | A | 0.35 | 0.222 | 0.041 | 6.88E-08 | -0.0709 | 0.032 | 0.028 | *SUB1* |
| rs1173719 | 5 | 32835981 | G | 0.48 | 0.177 | 0.039 | 5.40E-06 | -0.0542 | 0.028 | 0.056 | *SUB1* |
| rs12153415 | 5 | 32838491 | A | 0.22 | 0.195 | 0.046 | 2.78E-05 | -0.0332 | 0.018 | 0.061 | *MTMR12* |
| 5-32838703 | 5 | 32838703 | G | 0.48 | 0.177 | 0.039 | 5.40E-06 | -0.0542 | 0.028 | 0.056 | *SUB1* |
| **rs9292469** | **5** | **32840210** | **T** | **0.33** | **0.232** | **0.041** | **2.70E-08** | **-0.0416** | **0.016** | **0.013** | ***MTMR12*** |
| rs1173709 | 5 | 32841078 | C | 0.46 | 0.190 | 0.039 | 1.05E-06 | -0.0566 | 0.028 | 0.047 | *SUB1* |
| rs480882 | 5 | 32847023 | A | 0.50 | -0.182 | 0.039 | 4.46E-06 | 0.0691 | 0.026 | 0.009 | *SUB1* |

Bonferroni corrected for the number of probes tests across the two novel loci; p value threshold 0.05/24 = 2.1 x 10^-3^

**Supplementary Table 9: (Stage 3: Gene expression) Results from *cis*-eQTL analyses in primary osteoblasts for rs2697825 and SNPs in Linkage Disequilibrium (r^2^>0.8)**

|  |  |  |  |  | **HBM_AOGC GWAS Results** | | | **Osteoblast *cis*-eQTL Results** | | | |
| --- | --- | --- | --- | --- | --- | --- | --- | --- | --- | --- | --- |
| **rsID** | **Chr** | **Position** | **EA** | **EAF** | **Beta** | **SE** | **p value** | **Beta** | **SE** | **p value** | **Probe Gene** |
| rs17554294 | 11 | 14001535 | C | 0.10 | 0.327 | 0.074 | 9.59E-06 | -0.1140 | 0.032 | 6.62E-04 | *BTBD10* |
| rs72856939 | 11 | 14002826 | G | 0.10 | 0.324 | 0.073 | 1.02E-05 | na | na | na | na |
| rs147219855 | 11 | 14008932 | C | 0.10 | 0.323 | 0.073 | 1.03E-05 | -0.1141 | 0.032 | 6.47E-04 | *BTBD10* |
| rs138953345 | 11 | 14012004 | G | 0.10 | 0.324 | 0.073 | 1.02E-05 | -0.1141 | 0.032 | 6.41E-04 | *BTBD10* |
| rs12289489 | 11 | 14017988 | C | 0.10 | 0.318 | 0.073 | 1.47E-05 | -0.1142 | 0.032 | 6.30E-04 | *BTBD10* |
| rs4592401 | 11 | 14020157 | G | 0.10 | 0.324 | 0.073 | 8.91E-06 | -0.1143 | 0.032 | 6.13E-04 | *BTBD10* |
| rs16913542 | 11 | 14023610 | A | 0.10 | 0.319 | 0.072 | 1.04E-05 | -0.1147 | 0.032 | 5.66E-04 | *BTBD10* |
| rs9666215 | 11 | 14024134 | C | 0.10 | 0.319 | 0.072 | 1.01E-05 | -0.1347 | 0.038 | 2.69E-04 | *BTBD10* |
| rs7108768 | 11 | 14028519 | A | 0.10 | 0.329 | 0.071 | 4.71E-06 | -0.1147 | 0.032 | 5.66E-04 | *BTBD10* |
| rs72856961 | 11 | 14029738 | T | 0.10 | 0.325 | 0.072 | 6.74E-06 | -0.1147 | 0.032 | 5.66E-04 | *BTBD10* |
| rs10500789 | 11 | 14031713 | T | 0.10 | 0.330 | 0.072 | 4.55E-06 | -0.1147 | 0.032 | 5.66E-04 | *BTBD10* |
| rs56356622 | 11 | 14037738 | G | 0.10 | 0.313 | 0.071 | 1.13E-05 | -0.1147 | 0.032 | 5.66E-04 | *BTBD10* |
| rs17555076 | 11 | 14045203 | T | 0.10 | 0.308 | 0.071 | 1.59E-05 | -0.1147 | 0.032 | 5.66E-04 | *BTBD10* |
| rs2031014 | 11 | 14051977 | T | 0.11 | 0.303 | 0.069 | 1.35E-05 | -0.1147 | 0.032 | 5.66E-04 | *BTBD10* |
| rs4340032 | 11 | 14066059 | C | 0.10 | 0.300 | 0.070 | 1.89E-05 | -0.1147 | 0.032 | 5.66E-04 | *BTBD10* |
| rs16913550 | 11 | 14067374 | C | 0.10 | 0.301 | 0.070 | 1.84E-05 | -0.1144 | 0.032 | 5.66E-04 | *BTBD10* |
| rs61884837 | 11 | 14069721 | A | 0.11 | 0.297 | 0.070 | 2.42E-05 | -0.1144 | 0.032 | 5.66E-04 | *BTBD10* |
| rs55736726 | 11 | 14070423 | T | 0.10 | 0.299 | 0.070 | 1.99E-05 | -0.1144 | 0.032 | 5.66E-04 | *BTBD10* |
| rs11821106 | 11 | 14072602 | A | 0.10 | 0.299 | 0.070 | 2.00E-05 | -0.1147 | 0.032 | 5.66E-04 | *BTBD10* |
| rs7131289 | 11 | 14077871 | G | 0.10 | 0.291 | 0.070 | 3.57E-05 | -0.1147 | 0.032 | 5.65E-04 | *BTBD10* |
| rs2697822 | 11 | 14080686 | A | 0.17 | 0.298 | 0.057 | 2.27E-07 | -0.0668 | 0.024 | 6.35E-03 | *BTBD10* |
| rs2697823 | 11 | 14080925 | A | 0.17 | 0.298 | 0.057 | 2.43E-07 | -0.0589 | 0.024 | 1.54E-02 | *BTBD10* |
| **rs2697825** | **11** | **14089431** | **G** | **0.17** | **0.310** | **0.056** | **4.93E-08** | **-0.0635** | **0.025** | **1.47E-02** | ***BTBD10*** |
| rs2697826 | 11 | 14089440 | A | 0.17 | 0.310 | 0.056 | 4.93E-08 | -0.0635 | 0.025 | 1.47E-02 | *BTBD10* |
| rs72858616 | 11 | 14091354 | G | 0.11 | 0.306 | 0.069 | 1.04E-05 | -0.1147 | 0.032 | 5.66E-04 | *BTBD10* |

Bonferroni corrected for the number of probes tests across the two novel loci; p value threshold 0.05/24 = 2.1 x 10^-3^. na; not available

**Acknowledgments**

We would like to thank all study participants who provided DNA and clinical information.

Regarding the HBM study, we particularly thank staff at the Wellcome Trust Clinical Research Facility in Birmingham, Royal National Hospital for Rheumatic Diseases in Bath, Cambridge NIHR Biomedical Research Centre and Addenbrooke's Wellcome Trust Clinical Research Facility in Cambridge, Bone Research Unit in Cardiff, Musculoskeletal Research Unit in Bristol, NIHR Bone Biomedical Research Unit in Sheffield and the Brocklehurst Centre for Metabolic Bone Disease in Hull.

Regarding the AOGC study, we thank AOGC PIs; Eugene McCloskey (Sheffield, UK), Geoffrey C Nicholson (Geelong, Australia), Richard Eastell (Sheffield, UK), Richard L Prince (Perth, Australia), John A Eisman (Sydney, Australia), Graeme Jones (Hobart, Australia), Philip Sambrook (Sydney, Australia), Ian R Reid (Auckland, New Zealand), Elaine M Dennison (Southampton, UK), John Wark (Geelong, Australia). Furthermore, we thank Barbara Mason and Amanda Horne (Auckland) for patient recruitment; Judith Finigan (Sheffield, UK) for laboratory support and database support; Selina Simpson (Sheffield, UK) for DNA handling; Fatma Gossiel (Sheffield, UK) for DNA handling; Alison Steward and Lana Gibson (Aberdeen, UK) for patient recruitment; Katherine Kolk (Geelong, Australia); Janelle Rampellini (Perth, Australia) for patient recruitment; Jemma Christie (Melbourne, Australia) for patient recruitment; Helen Steane (Hobart, Australia) for patient recruitment; Denia Mang and Ruth Toppler for DNA extraction, DNA handling, and database support (Dubbo/Sydney, Australia); Kate Lowings (Brisbane, Australia) for patient recruitment; and Marieke Brugmans and Leanne Brookes (Brisbane, Australia) for DNA preparation and genotyping. We thank Ms Linda Bradbury (Brisbane, Australia) for support with ethics, governance and recruitment.

The HBM study was supported by the UK NIHR CRN (portfolio number 5163); supporting CLRNs included Birmingham and the Black Country, London South, Norfolk & Suffolk, North and East Yorkshire and Northern Lincolnshire, South Yorkshire, Surrey & Sussex, West Anglia and Western.

The AOGC also received funding from the Australian Cancer Research Foundation and Rebecca Cooper Foundation (Australia). MAB was funded by a National Health and Medical Research Council (Australia) Principal Research Fellowship and ELD was funded by a National Health and Medical Research Council (Australia) Career Development Award (569807). IR is supported by the Health Research Council of New Zealand. The OPUS study was supported by Sanofi-Aventis, Eli Lilly, Novartis, Pfizer, Proctor&Gamble Pharmaceuticals and Roche. The Sydney Twin Study was supported by the National Health and Medical Research Council, Australia. The Dubbo Osteoporosis Epidemiology Study was supported by the Australian National Health and Medical Research Council, MBF Living Well foundation, the Ernst Heine Family Foundation and from untied educational grants from Amgen, Eli Lilly International, GE-Lunar, Merck Australia, Novartis, Sanofi-Aventis Australia and Servier. The Hertfordshire Cohort Study was supported by grants from the Medical Research Council UK & Arthritis Research UK. The Geelong Osteoporosis Study was funded by grants from the Victorian Health Promotion Foundation and the Geelong Region Medical Research Foundation, and the National Health and Medical Research Council, Australia (project grant 628582). The Oxford Osteoporosis Study was funded by Action Research UK.

**References**

1. Gregson CL, Hardcastle SA, Cooper C, Tobias JH. Friend or foe: high bone mineral density on routine bone density scanning, a review of causes and management. Rheumatology. 2013;52(6):968-85.

2. Kellgren J, Lawrence J. Radiological assessment of osteo-arthrosis. Ann Rheum Dis. 1957;16(4):494-502.

3. Gregson CL, Steel S, Yoshida K, Reid DM, Tobias JH. An investigation into the impact of osteoarthritic changes on bone mineral density measurements in patients with High Bone Mass. ASBMR 30th Annual Meeting, Montreal 2008;SA257.

4. Hansen KE, Binkley N, Christian R, Vallarta-Ast N, Krueger D, Drezner MK, et al. Interobserver reproducibility of criteria for vertebral body exclusion. J Bone Miner Res. 2005;20(3):501-8.

5. White J, Yeats A, Skipworth G. Tables for Statisticians: Stanley Thornes, Cheltenham; 1979.

6. Gregson CL, Steel SA, O'Rourke KP, Allan K, Ayuk J, Bhalla A, et al. 'Sink or swim': an evaluation of the clinical characteristics of individuals with high bone mass. Osteo Int. 2012;23(2):643-54.

7. The 59th General Assembly Seoul. World Medical Assembly Declaration of Helsinki - Ethical Principles for Medical Research Involving Human Subjects. Seoul, Korea 2008 [

8. Henry M, Pasco J, Korn S, Gibson J, Kotowicz M, Nicholson G. Bone mineral density reference ranges for Australian men: Geelong Osteoporosis Study. Osteoporosis International. 2010;21(6):909-17.

9. Bahlo M, Stankovich J, Danoy P, Hickey PF, Taylor BV, Browning SR, et al. Saliva-derived DNA performs well in large-scale, high-density single-nucleotide polymorphism microarray studies. Cancer Epidemiol Biomarkers Prev. 2010;19(3):794-8.

10. Gluer CC, Eastell R, Reid DM, Felsenberg D, Roux C, Barkmann R, et al. Association of five quantitative ultrasound devices and bone densitometry with osteoporotic vertebral fractures in a population-based sample: the OPUS Study. J Bone Miner Res,. 2004;19(5):782-93.

11. McCloskey E, Selby P, Davies M, Robinson J, Francis RM, Adams J, et al. Clodronate reduces vertebral fracture risk in women with postmenopausal or secondary osteoporosis: results of a double-blind, placebo-controlled 3-year study. J Bone Miner Res,. 2004;19(5):728-36.

12. Simons LA, McCallum J, Simons J, Powell I, Ruys J, Heller R, et al. The Dubbo study: an Australian prospective community study of the health of elderly. Australian and New Zealand journal of medicine. 1990;20(6):783-9.
