# Supplementary material for: Genome-wide association study of extreme high bone mass: Contribution of common genetic variation to extreme BMD phenotypes and potential novel BMD-associated genes
Source: Bone. 2018 Sep;114:62–71. doi: 10.1016/j.bone.2018.06.001 (PMC6086337; doi:10.1016/j.bone.2018.06.001)
Supplement: Supplementary file 2 — Supplementary figures 1 to 5 [file mmc2.pdf]

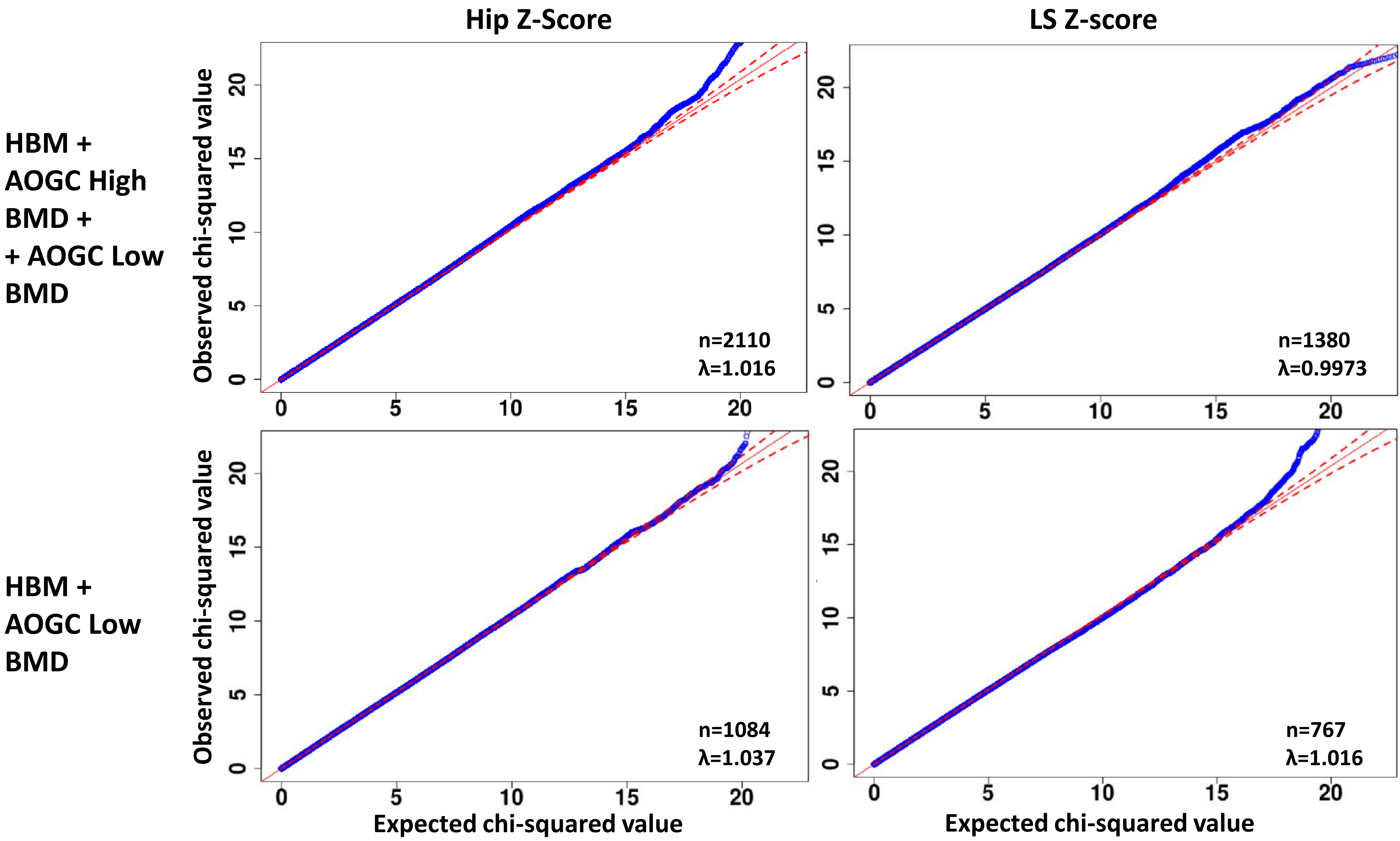

Supplementary Fig. 1

QQ plots, with 95% confidence internals shown (red), for the HBM, AOGC High BMD cases and AOGC Low BMD controls quantitative trait genome-wide association studies of Total Hip and Lumbar Spine BMD Z-Scores, adjusted for age, age2 and center

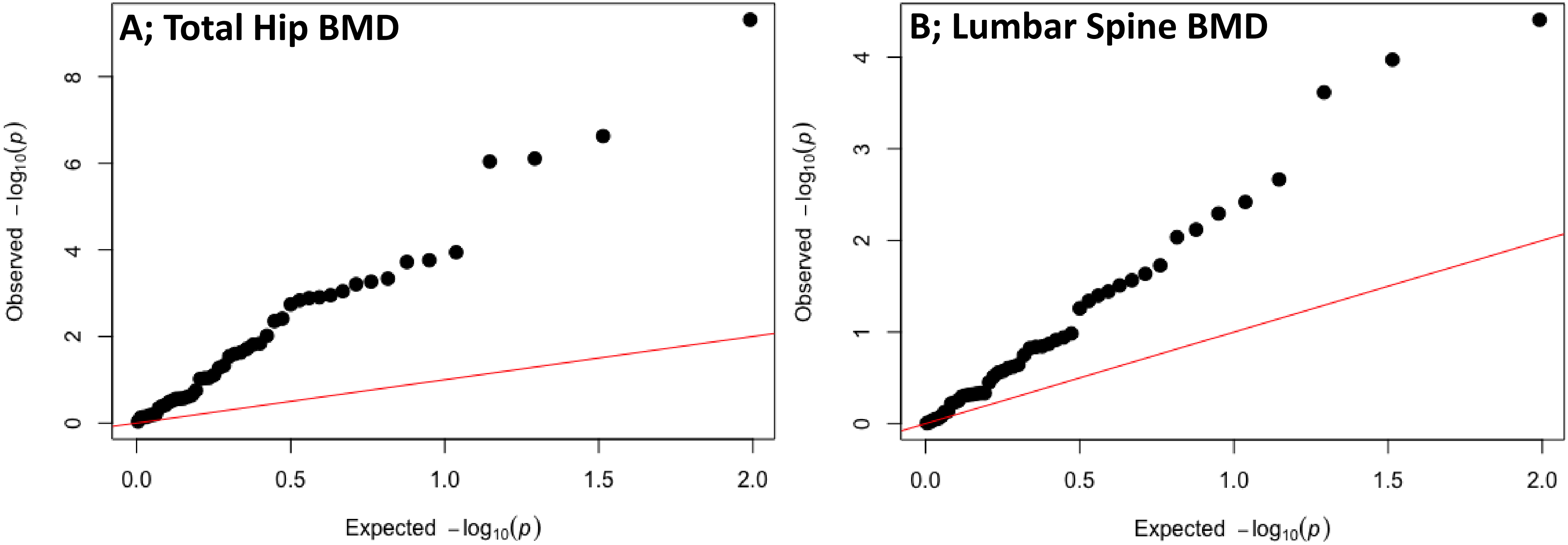

Supplementary Fig. 2

QQ plot for p values for 49 established BMD-associated loci in GWAS of (A) TH BMD and (B) LS BMD in HBM cases, AOGC high BMD cases and AOGC low BMD controls (adjusted for age age^2, center)

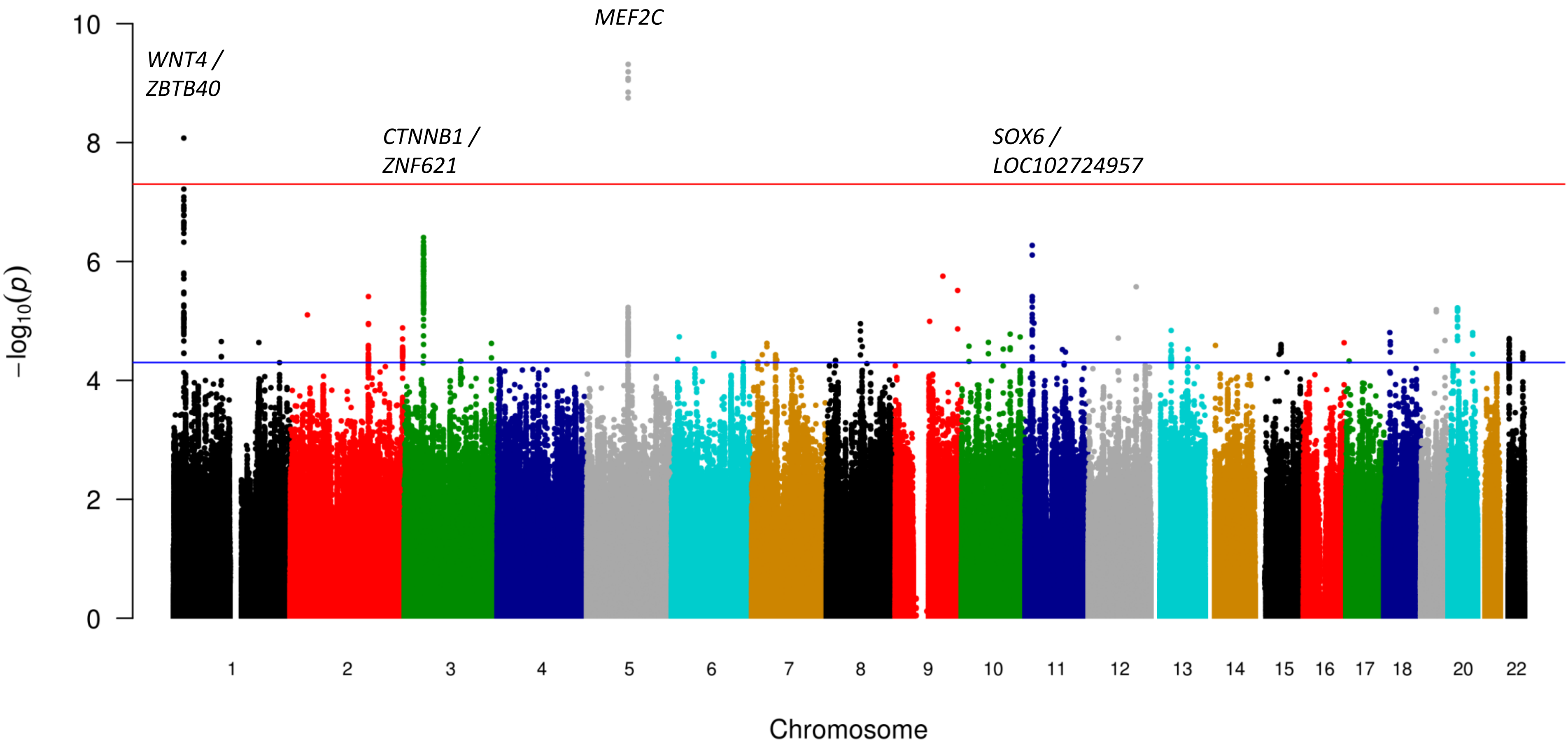

Supplementary Fig. 3a

Manhattan plot for the HBM, AOGC High BMD cases and AOGC Low BMD controls genome-wide association study of Total Hip BMD Z-Score, adjusted for age, age2 and center

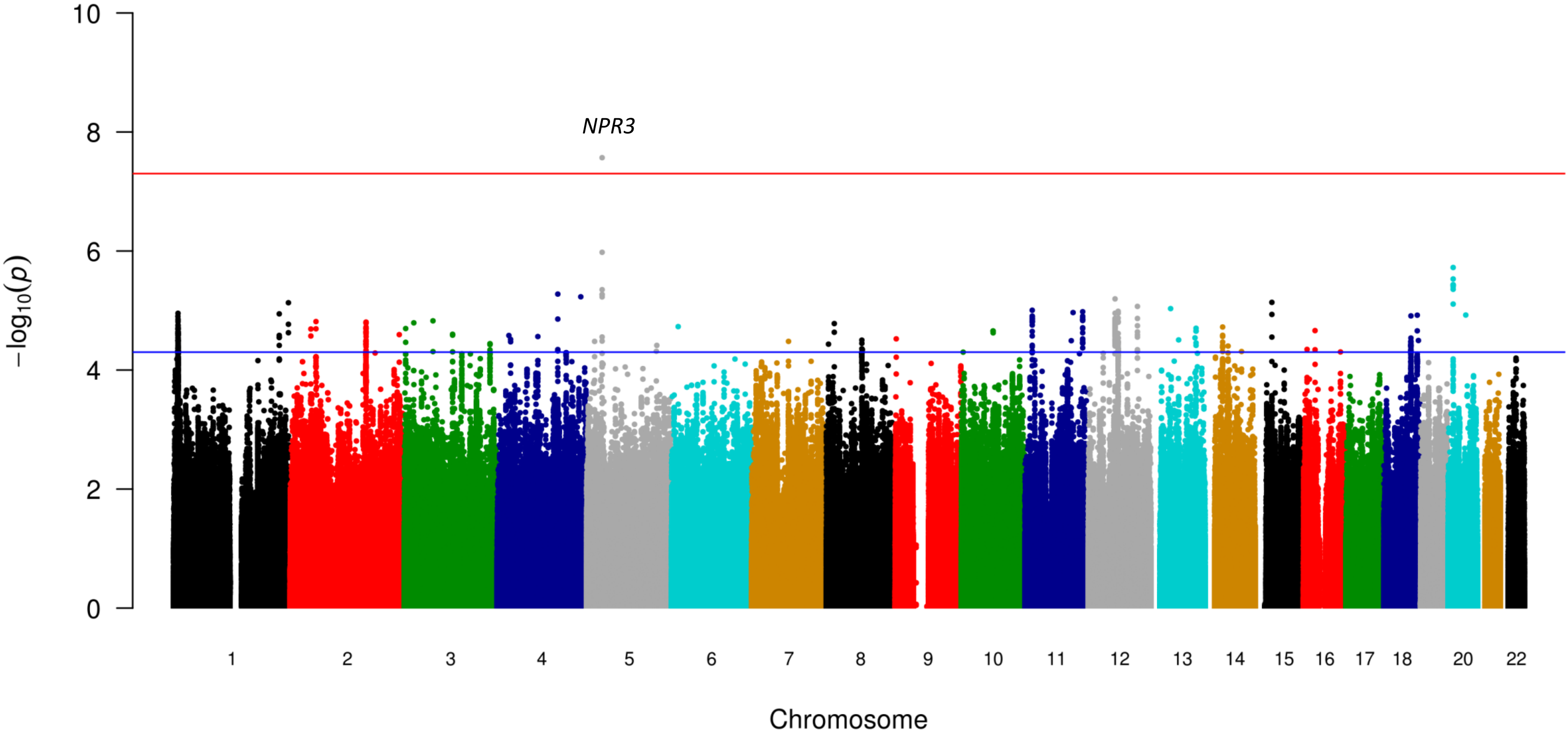

Supplementary Fig. 3b
Manhattan plot for the HBM, AOGC High BMD cases and AOGC Low BMD controls genome-wide association study of Lumbar Spine BMD Z-Score, adjusted for age, age2 and center

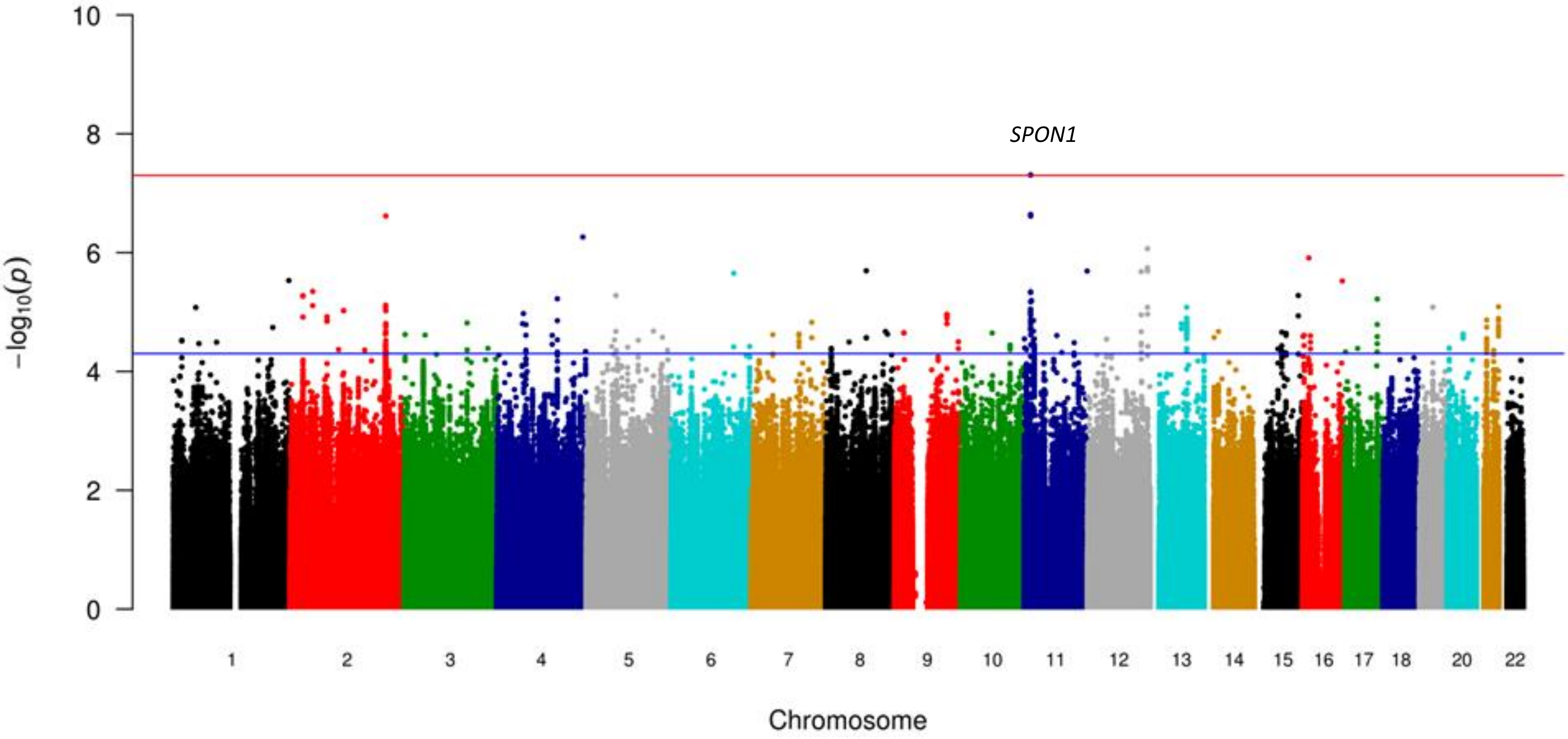

Supplementary Fig. 3c
Manhattan plot for the HBM cases and AOGC Low BMD controls genome-wide association study of Total Hip BMD Z-Score, adjusted for age, age2 and center

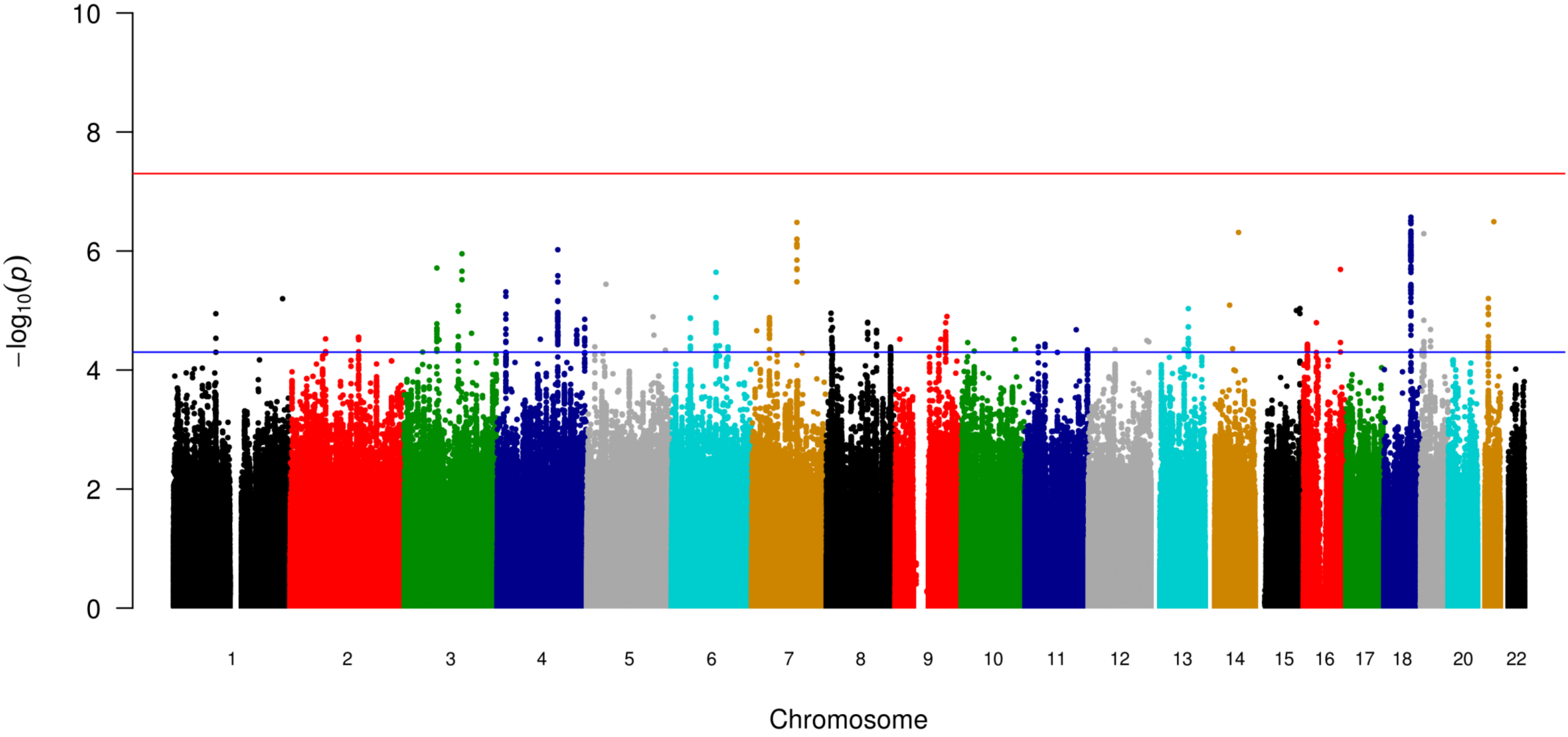

Supplementary Fig. 3d
Manhattan plot for the HBM cases and AOGC Low BMD controls genome-wide association study of Lumbar Spine BMD Z-Score, adjusted for age, age2 and center

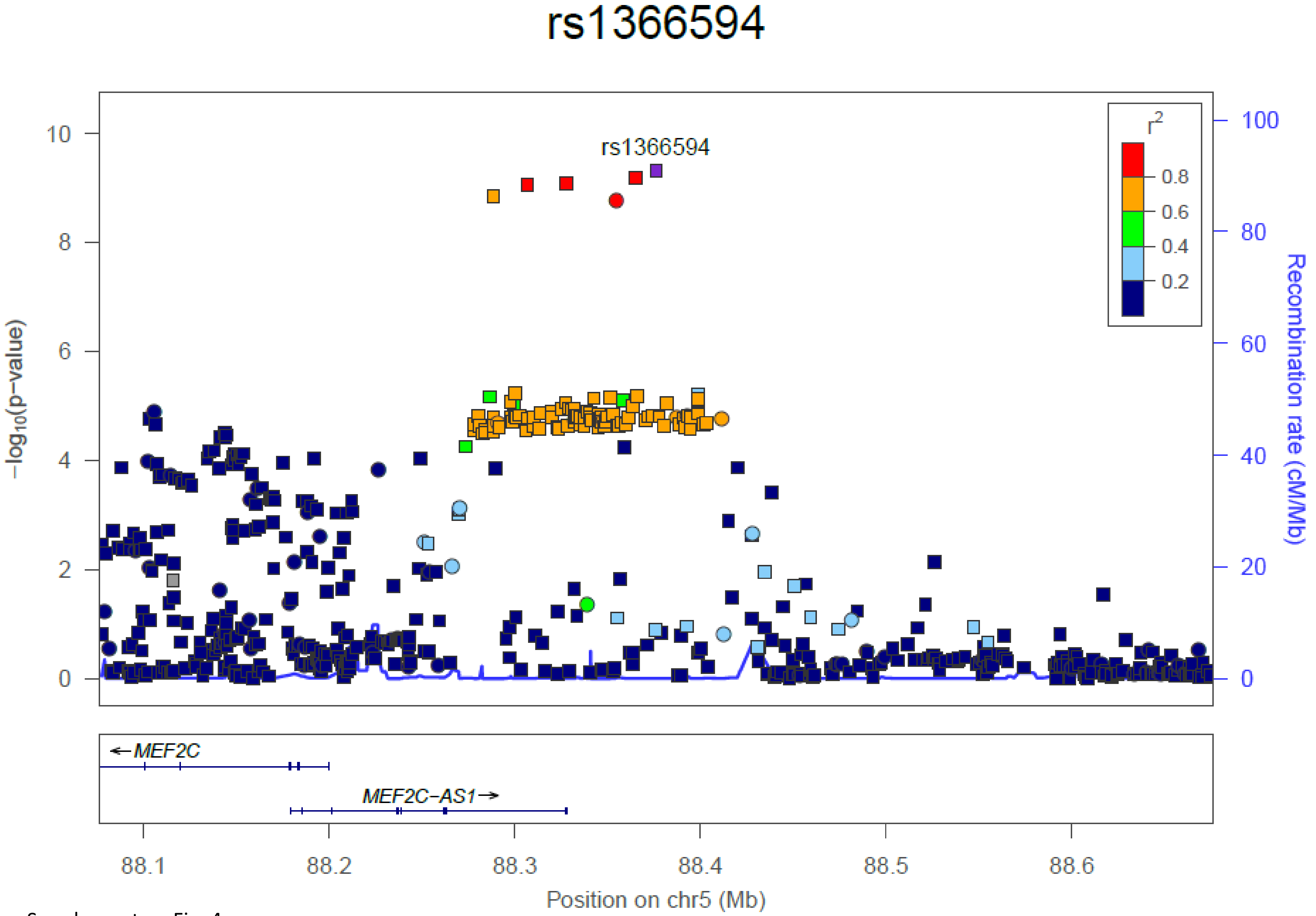

Supplementary Fig. 4a

MEF2C regional association plot of the HBM, AOGC High BMD cases and AOGC Low BMD controls genome-wide association study of Total Hip BMD Z-Score, adjusted for age, age2 and center (300kb either side of rs1366594 shown). Square symbols indicate imputed SNPs; circles indicate those genotyped.

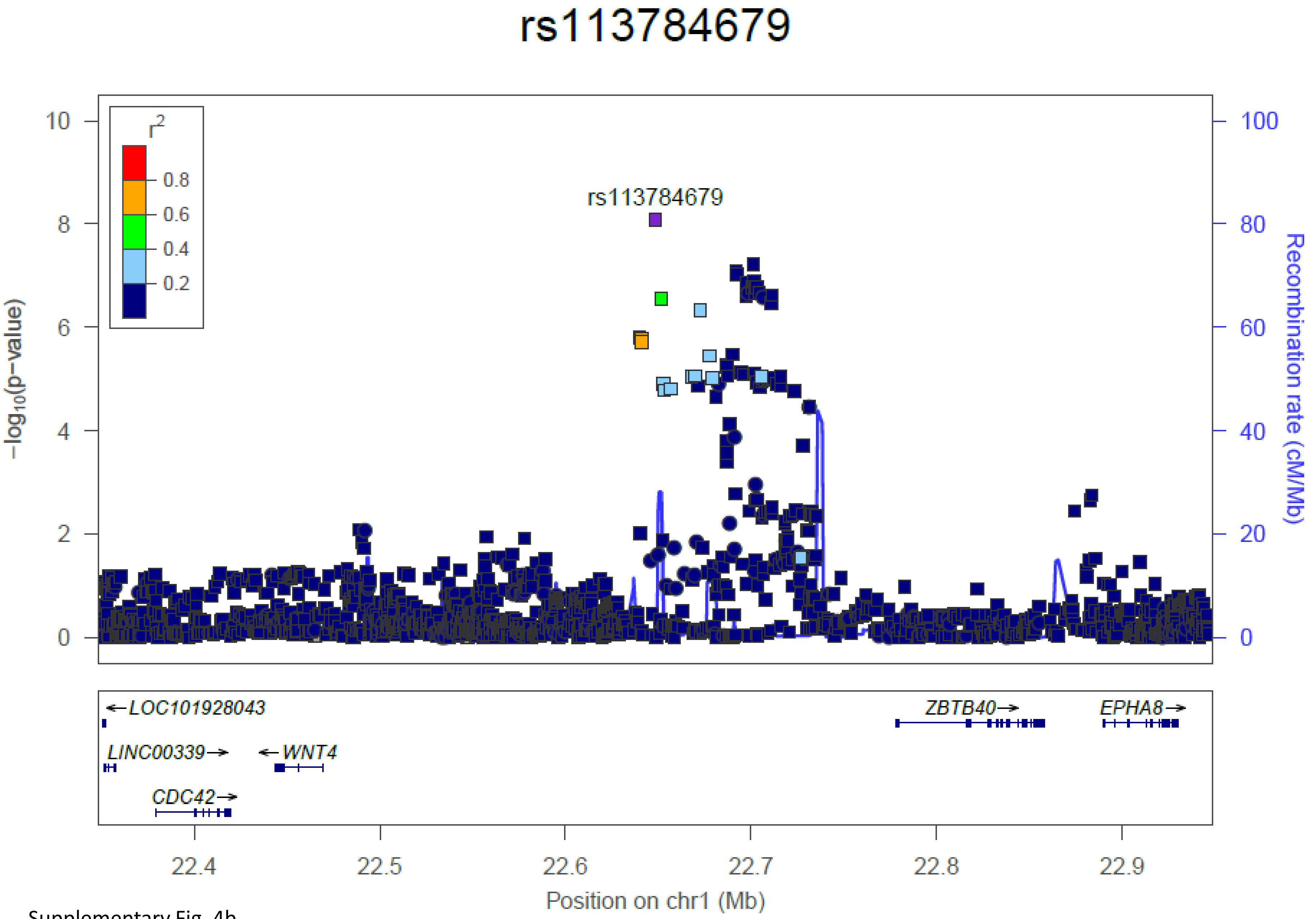

Supplementary Fig. 4b

WNT4/ZBTB40 regional association plot of the HBM, AOGC High BMD cases and AOGC Low BMD controls genome-wide association study of Total Hip BMD Z-Score, adjusted for age, age2 and center (1000kb either side of rs113784679 shown). Square symbols indicate imputed SNPs; circles indicate those genotyped.

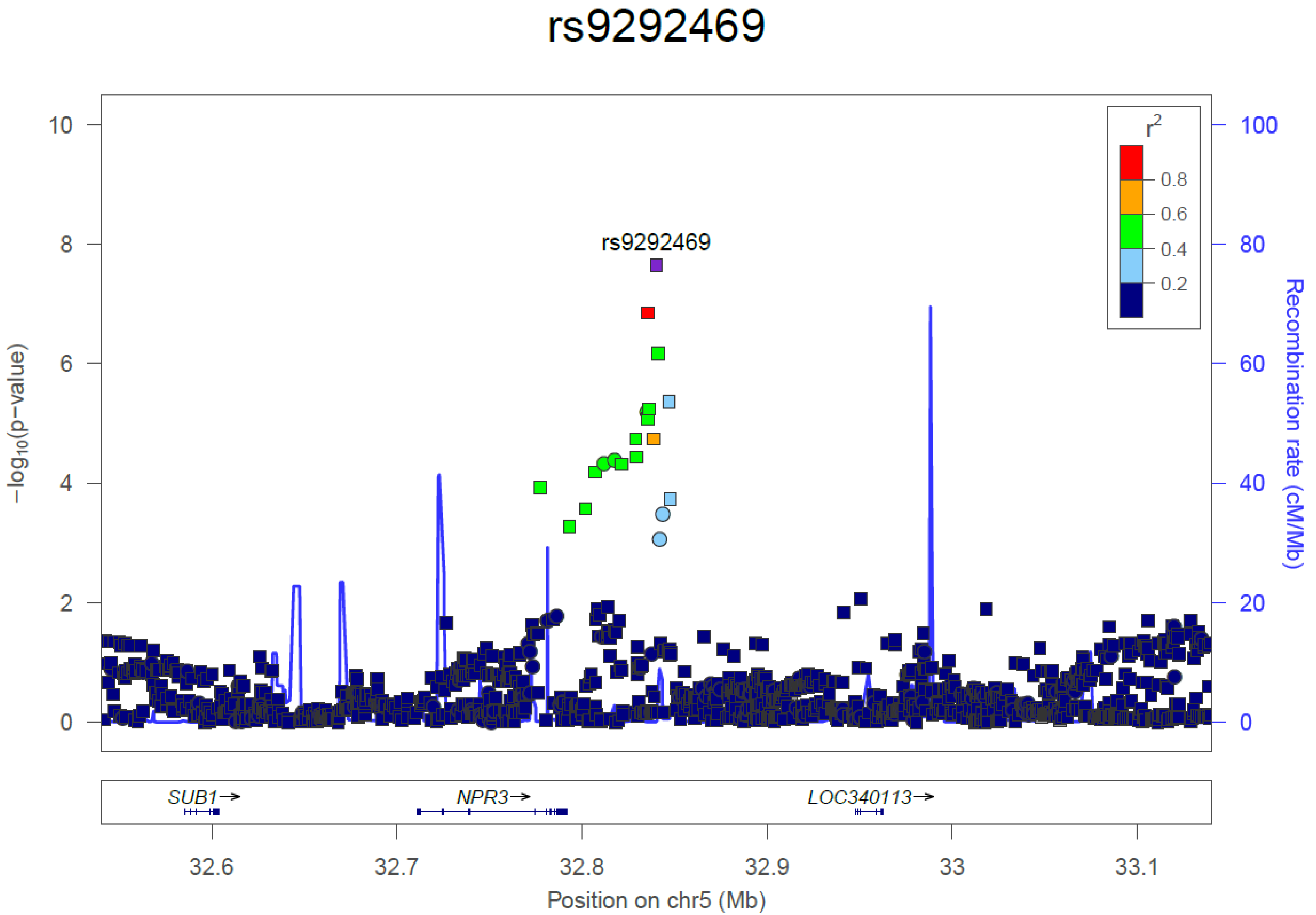

Supplementary Fig. 5
NPR3 regional association plot of the HBM, AOGC High BMD cases and AOGC Low BMD controls genome-wide association study of Lumbar Spine BMD Z-Score, adjusted for age, age2, center **AND HEIGHT** (300kb either side of rs9292469 shown)
